# Gaze behavior and cognitive states during fingerprint target group localization

**DOI:** 10.1186/s41235-019-0160-9

**Published:** 2019-04-05

**Authors:** R. Austin Hicklin, Bradford T. Ulery, Thomas A. Busey, Maria Antonia Roberts, JoAnn Buscaglia

**Affiliations:** 1 Noblis, 2002 Edmund Halley Drive, Reston, VA 20191 USA; 2 Department of Psychology, Indiana University, Bloomington, IN 47405 USA; 3 Latent Print Support Unit, Federal Bureau of Investigation Laboratory Division, 2501 Investigation Parkway, Quantico, VA 22135 USA; 4 Counterterrorism and Forensic Science Research Unit, Federal Bureau of Investigation Laboratory Division, 2501 Investigation Parkway, Quantico, VA 22135 USA

**Keywords:** Eye tracking, Forensics, Latent fingerprints

## Abstract

**Background:**

The comparison of fingerprints by expert latent print examiners generally involves repeating a process in which the examiner selects a small area of distinctive features in one print (a target group), and searches for it in the other print. In order to isolate this key element of fingerprint comparison, we use eye-tracking data to describe the behavior of latent fingerprint examiners on a narrowly defined “find the target” task. Participants were shown a fingerprint image with a target group indicated and asked to find the corresponding area of ridge detail in a second impression of the same finger and state when they found the target location. Target groups were presented on latent and plain exemplar fingerprint images, and as small areas cropped from the plain exemplars, to assess how image quality and the lack of surrounding visual context affected task performance and eye behavior. One hundred and seventeen participants completed a total of 675 trials.

**Results:**

The presence or absence of context notably affected the areas viewed and time spent in comparison; differences between latent and plain exemplar tasks were much less significant. In virtually all trials, examiners repeatedly looked back and forth between the images, suggesting constraints on the capacity of visual working memory. On most trials where context was provided, examiners looked immediately at the corresponding location: with context, median time to find the corresponding location was less than 0.3 s (second fixation); however, without context, median time was 1.9 s (five fixations). A few trials resulted in errors in which the examiner did not find the correct target location. Basic gaze measures of overt behaviors, such as speed, areas visited, and back-and-forth behavior, were used in conjunction with the known target area to infer the underlying cognitive state of the examiner.

**Conclusions:**

Visual context has a significant effect on the eye behavior of latent print examiners. Localization errors suggest how errors may occur in real comparisons: examiners sometimes compare an incorrect but similar target group and do not continue to search for a better candidate target group. The analytic methods and predictive models developed here can be used to describe the more complex behavior involved in actual fingerprint comparisons.

**Electronic supplementary material:**

The online version of this article (10.1186/s41235-019-0160-9) contains supplementary material, which is available to authorized users.

## Significance statement

Visual localization is a critical task within the fingerprint comparison process, in which the fingerprint examiner analyzes and memorizes a specific area of detail in one fingerprint and searches for the corresponding area in another fingerprint. This study isolates individual localization tasks, and details how eye-gaze behavior can be used to describe and analyze how fingerprint examiners perform localization.

## Background

This study is the first detailed evaluation of localization in fingerprint comparison. Latent fingerprint comparison is critically important within the criminal justice system. A variety of studies have shown that the accuracy and reliability of conclusions by fingerprint examiners are imperfect (e.g., Neumann, Champod, Yoo, Genessay, & Langenburg, 2013; Pacheco, Cerchiai, & Stoiloff, 2014; Ulery, Hicklin, Buscaglia, & Roberts, 2011). Such studies show that some examiners (rarely) make erroneous identifications, erroneous exclusions are much more prevalent than erroneous identifications, and examiners often are inconclusive on comparisons that resulted in identifications from other examiners. Understanding why such errors and disagreements occur requires detailed evaluation of fingerprint examiners. Here we evaluate a critical subtask of fingerprint comparison in order to gain a greater understanding of how experts compare fingerprints, and why they differ.

Trained latent print examiners visually compare fingerprints to determine whether the prints share enough detail in agreement to conclude that they came from the same source. Currently, human experts (not computers) make the final conclusions on latent print comparisons, using their expertise rather than a quantitative standard to determine if the information content is sufficient to make a decision. The entire fingerprint examination process involves a number of perceptual and memory-based mechanisms, but one of the fundamental subtasks is *target group localization*: selecting a small area of distinctive features (a *target group*), analyzing and memorizing that specific area of detail in one image, and searching for the corresponding area in another image (Ashbaugh, 1999; Expert Working Group on Human Factors in Latent Print Analysis, 2012; National Institute of Justice, 2011). During comparison, the examiner iteratively selects and compares additional target groups, accumulating evidence to make a decision as to whether the two impressions came from the same or different sources. One challenge when working with fingerprint experts is that there are substantial differences among examiners: previous work has shown that latent print examiners vary significantly in their determinations (Pacheco et al., 2014; Ulery et al., 2011; Ulery, Hicklin, Buscaglia, & Roberts, 2012), as well as in the features used as the bases for their decisions (Langenburg, Champod, & Genessay, 2012; Neumann et al., 2013; Ulery, Hicklin, Roberts, & Buscaglia, 2014, 2016). Because target group localization is at the core of the fingerprint comparison process, understanding localization should provide insight into that process, and provide a basis for understanding how and why examiners vary in their conclusions. The fingerprint discipline can use such information in improving processes and minimizing sources of errors — and the broader forensic science and legal communities may use such information to more completely understand the reliability of conclusions by fingerprint examiners.

### Deconstructing tasks into constituent elements

Isolating specific subtasks can make the study of complex visual behavior tractable (Hayhoe, 2000; Johnson, Sullivan, Hayhoe, & Ballard, 2014). A full fingerprint comparison involves the localization of multiple target groups, which in aggregate provide support for (or against) a source determination. Each time a target group is selected, those features are placed into visual working memory (Baddeley, 1986), which is used when comparing against details in the other print. Establishing correspondence relies on the quality of the impressions and the distinctiveness of the configuration of the features in the target group. Eye behavior is constrained by the small features (minutiae), the narrow visual field of the fovea, and the capacity and persistence of visual working memory. The process of finding an area that corresponds to a selected target group typically requires multiple fixations on the latent impression, and then multiple fixations on the exemplar impression (Busey et al., 2011; Busey, Yu, Wyatte, & Vanderkolk, 2013; Busey, Swofford, Vanderkolk, & Emerick, 2015).

It may be instructive to compare this target group localization task to a “Where’s Waldo” task (e.g., Credido, Teixeira, Reis, Moreira, & Andrade, 2012) using elements of Kundel’s deconstruction of detection (Kundel, Nodine, & Carmody, 1978). These elements include *orientation* (“what do I have to work with?” — assessing the overall image); *scanning* (“where is it?” — looking for potential locations); *recognition* (“is this it?” — recognizing as a potential target when seen); and *decision* (“am I sure?” — deciding whether it is the correct target). A number of authors have extended Kundel’s idea of inferring underlying cognitive states from eye-gaze behavior and it has proven to be useful when deconstructing a task into constituent elements (e.g., Kardan, Berman, Yourganov, Schmidt, & Henderson, 2015; Land, Mennie, & Rusted, 1999; Marshall, 2007). Ballard, Hayhoe, and Pelz (1995) used a block copying task to identify when participants relied on working memory and when they chose to externalize this memory to the physical world. Later work by Hayhoe (2000) extended this approach and identified specific visual routines as well as the information used by each when performing a block copying or driving task. Similar cognitive states may underlie fingerprint target localization as examiners progress from a search phase to a decision phase, and there may be characteristic eye behaviors associated with each phase.

### The role of context

In the *scanning* state of “Where’s Waldo,” knowing what Waldo looks like does not provide any information regarding where to look in the image because context is deliberately minimized or eliminated by the artist. With a large fingerprint impression, however, the overall ridge flow and pattern type provides extensive contextual information useful in localizing a target group. This contextual information need not be explicitly provided: skilled examiners often can look at a portion of a fingerprint and infer the approximate location on the finger from its local ridge details. In addition, context may improve the interpretation of the details (Barenholtz, 2014). In *recognition*, the *Where’s Waldo* pictures contain similar-looking decoys that slow the process of finding the target; as we will see, fingerprints may also contain such decoys. After Waldo has been recognized, it is generally trivial to decide that it is Waldo and not a decoy; however, two impressions of the same finger may vary considerably in appearance and quality, so the process of deciding whether two target groups correspond is often not trivial. Relational information from macro features, such as the core or delta, may help the examiner eliminate some decoys, whereas relational information is made deliberately uninformative in the *Where’s Waldo* task. Thus, whereas *Where’s Waldo* can be viewed as a fairly traditional visual search task, fingerprint comparisons may involve a tight interplay between the appearance of individual features and the broader context in which they appear.

Natural scenes may serve as a good model for fingerprints because they also contain structural information that provides context that guides the interpretation of individual features. Of the few studies that have addressed the eye-gaze behavior of fingerprint examiners (Busey et al., 2011, 2013, 2015), none has addressed the effect of context. However, working with structured displays, Chun and Jiang (1998, 1999) found that spatial configuration information can indirectly guide search to the target because the visual covariation of the structure of a display is learned; similar experience may help fingerprint examiners infer the general location of a region from a partial print (e.g., deltas are generally found below the core). Neider and Zelinsky (2006) addressed the role of context in natural scene perception using target images (blimps and cars) that were either presented in their correct context or in an incorrect context (sky or ground). They argued that contextual information creates expectations that guide search behavior by biasing searches toward target-consistent regions. Similar effects were modeled by Oliva and Torralba (2007) to demonstrate how the scene structure and prior knowledge of the world can be used to construct a saliency map that adds contextual influences to bottom-up processes. The general consensus of the literature is that objects presented in context are recognized faster and more accurately than objects that are not, and that context might influence the search and localization of a target feature through top-down mechanisms (but see Heeger, 2017 for a bi-directional approach).

Fingerprint impressions contain local pattern information that could provide the basis for expectations that guide search to regions that are more likely to contain a target region. Similar biasing was identified by Pomplun (2006), who asked participants to first encode a small target and then search for that target in a naturalistic image such as a clock face or landscape. His results suggested that subjects were sensitive to the similarity of the target and various regions of the image, and quantified this using a measure termed *saccadic selectivity*. Observers tended to saccade to regions of the searched image that bear similarity to the target, although this can depend on the relevant dimension in a particular task. For example, if color is irrelevant, observers will shift to other dimensions, such as shape, as the basis for their saccades. Based on his results, we would expect clusters of distributions on regions that are similar in appearance to our target region, although surrounding spatial context may help eliminate some decoy regions.

### Study goals

In order to characterize examiner behavior when localizing a target group in a comparison image and to determine how that behavior is modulated by context, we designed an experiment to assess how examiners accomplish the localization task as it occurs during fingerprint comparison, with these goals:

- *Assess the effects of context:* our first goal is to assess the effects of visual context on the eye behavior of examiners, and what this reveals about the perceptual and cognitive mechanisms that support localization
- *Deconstruct the localization task:* our second goal is to assess whether we can deconstruct the localization task, mapping observable eye-gaze behavior to underlying cognitive states such as searching or deciding
- *Develop analytic tools:* our third goal is to develop tools to describe and analyze eye-gaze behavior in fingerprint comparison tasks

To accomplish these goals, we proceed in several stages. First, in the “Describing eye-gaze behavior in localization” section, we explore a variety of descriptive methods: these methods and the resulting findings have both theoretical and applied interest to the scientific and forensic communities. In the “Characterizing eye-gaze behavior in localization” section, we define methods to categorize and characterize eye-gaze behavior during localization. In the “Inferring cognitive states” section, we then use these methods to infer underlying cognitive states from the overt behaviors.

Looking beyond the current study, the longterm purpose of this work will be to use the results, lessons learned, methods, and metrics from this experiment as the basis for ongoing and future analyses of fingerprint comparisons, specifically in order to understand the extent to which errors and differences in conclusions can be explained by differences in behavior.

## Methods

**Fig. 1:**
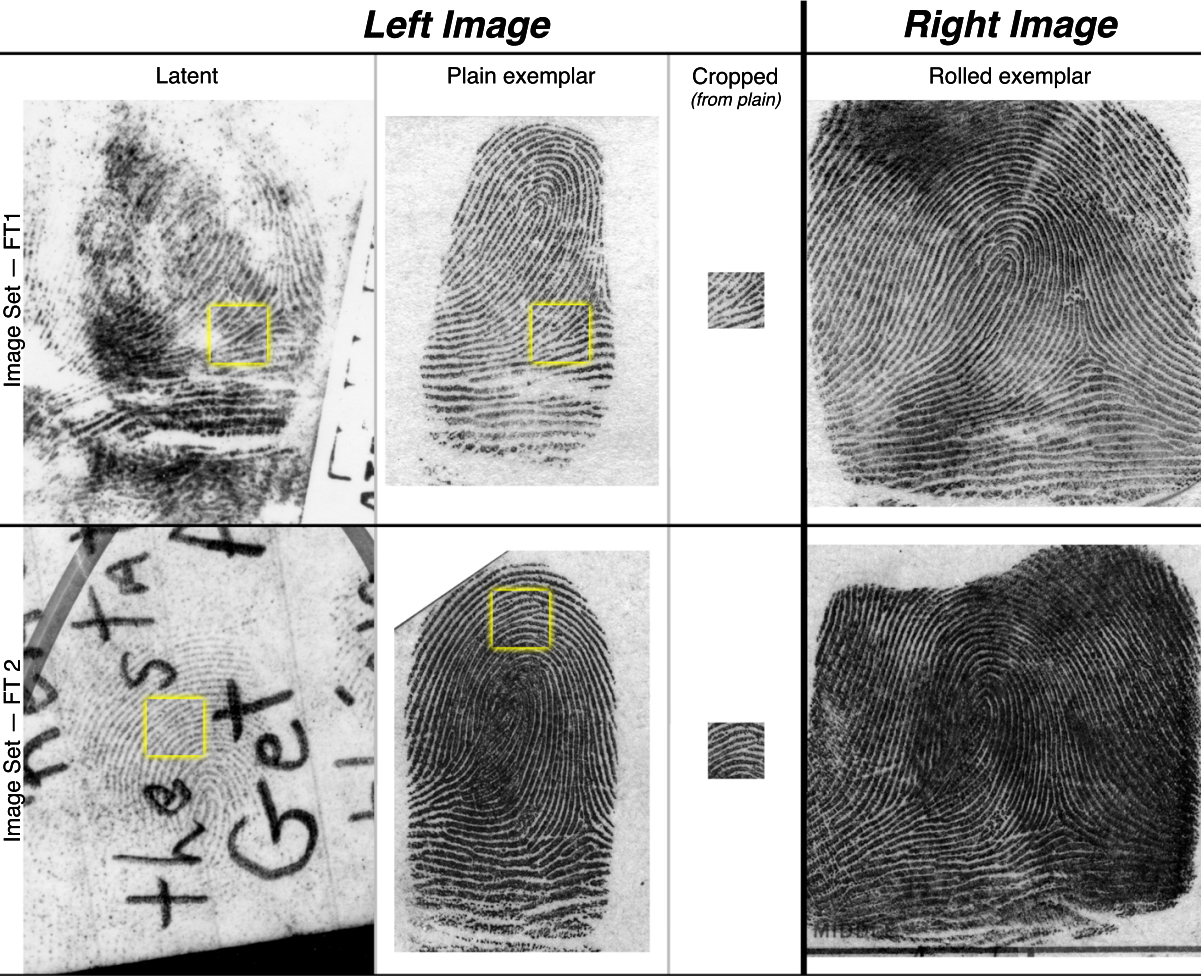
Two example sets of images used in find-the-target tasks. In each trial, one of the left images (Latent, Plain, or Cropped) was paired with the right image (mated exemplar). The yellow squares indicating the target areas were in the images shown to the participants. All images are at the same scale. Target area is 3.8 mm × 3.8 mm. Latent images (left column) are slightly cropped for publication. See Additional file 1: Appendix SI-3 for the other image sets

Preselected target groups were presented in one of three conditions: in the context of a typical latent fingerprint; in the context of a high-clarity plain (not rolled) exemplar^1^; or as a small area cropped from the plain exemplar (without surrounding context) (Fig. 1). Examiners then localized the designated target group in a second impression that the examiners were told was from the same source (mated); the mated impression was the same for all three conditions. The use of preselected target groups and mated impressions allows us to isolate localization behavior from other elements such as target selection and the decision process that results in a conclusion. This task was part of a larger experiment that involved other tasks such as counting and tracing ridges, as well as easy and difficult comparisons with realistic latent prints.

An Eyelink 1000 eye-tracker was used to track eye movement while examiners analyzed and compared fingerprint images on computer screens. Instructions to participants were as follows:

“Find the target group — You will be shown an image on the left with a target group already identified/indicated (a square about eight ridges on a side). The target group may be shown as an outlined area within a full exemplar or latent, or as a small cropped area shown by itself. You will then be shown a rolled exemplar from the same source (same finger, same subject, with the same orientation). Find the indicated target group, and when you are confident that you have found it, say DONE. The target group will always be present. (The purpose is so that we can understand in isolation the behavior of your eyes when memorizing a group of features, and searching for that group of features — which we expect to see often in standard comparisons. We are not trying to trick you (the source is always there): we are trying to see how you memorize and search for a target group.)”

### Fingerprint images

Eight *image sets* were selected from the “Ground Truth” dataset distributed with the Federal Bureau of Investigation’s (FBI) Universal Latent Workstation (ULW, n.d.). Each image set was constructed from three impressions of the same finger (latent, plain (flat) exemplar, rolled exemplar) scanned at 39.4 pixels per millimeter (ppmm) — note that the images were scanned at 1000 pixels per inch in accordance with the prevailing standard (ANSI/NIST-ITL, 2013), and therefore metric equivalents are rounded. When presented to an examiner, the image on the left was either (a) the latent image, (b) the plain exemplar image, or (c) a small area cropped from the plain exemplar image; the mated, high-clarity rolled exemplar was always shown on the right side of the screen. The images from two image sets are shown in Fig. 1.

A small area of each finger was selected as the “*target group*.” This target group was shown outlined in yellow on the latent and plain image; the cropped image was identical to the target group outlined on the plain image but without the surrounding context. Each target group area was a 150 × 150 pixel square (3.8 mm × 3.8 mm; approximately eight ridges across assuming an average ridge-ridge distance of 0.56 mm). Because of the plasticity of the skin, the areas on the latent and plain images were not strictly identical. Thus, eight image sets (FT1–FT8) were defined; within each set three image pairs were defined, with the left image varying based on *the task type* (*latent, plain, cropped*), but with the same corresponding rolled exemplar used as the right image. We refer to image pairs as FT1_Latent_, FT1_Plain_, FT1_Crop_, etc. The image in the plain and cropped tasks was identical except for the visual context included in the plain tasks, so that comparing plain to cropped would isolate the effect of context.

The target areas were selected to provide a moderate amount of pattern-level information, so that the task was tractable but not obvious. We considered selecting low-information target groups (e.g., areas where ridges are relatively parallel with few minutiae, or targets cropped from the latent prints), but rejected that because it would change the scenario to one where the participants might not have enough information to complete the task. Conversely, we also avoided highly distinctive areas: the reason that we stopped using target groups FT7 and FT8 was because they were too obvious (clearly core and delta formations, respectively).

In order to isolate the specific task of finding the target and determine the role of context, we deliberately only used fingerprints from the same source (mated image pairs), and told the examiners that they were mated. Using only mated image pairs was necessary: if examiners were also deciding whether the images were mated, they presumably would have compared in detail regions outside the target group. This would have turned our task into a full examination, instead of the intended subset of the comparison process.

When assessing whether a fixation is considered in the target area, we add a margin of 30 pixels (just over one ridge width) to the 150 × 150 pixel target to allow for factors such as eye-tracker measurement imprecision (e.g., calibration), foveal field of view, distortion, and skin elasticity.

### Data collection setup

**Fig. 2:**
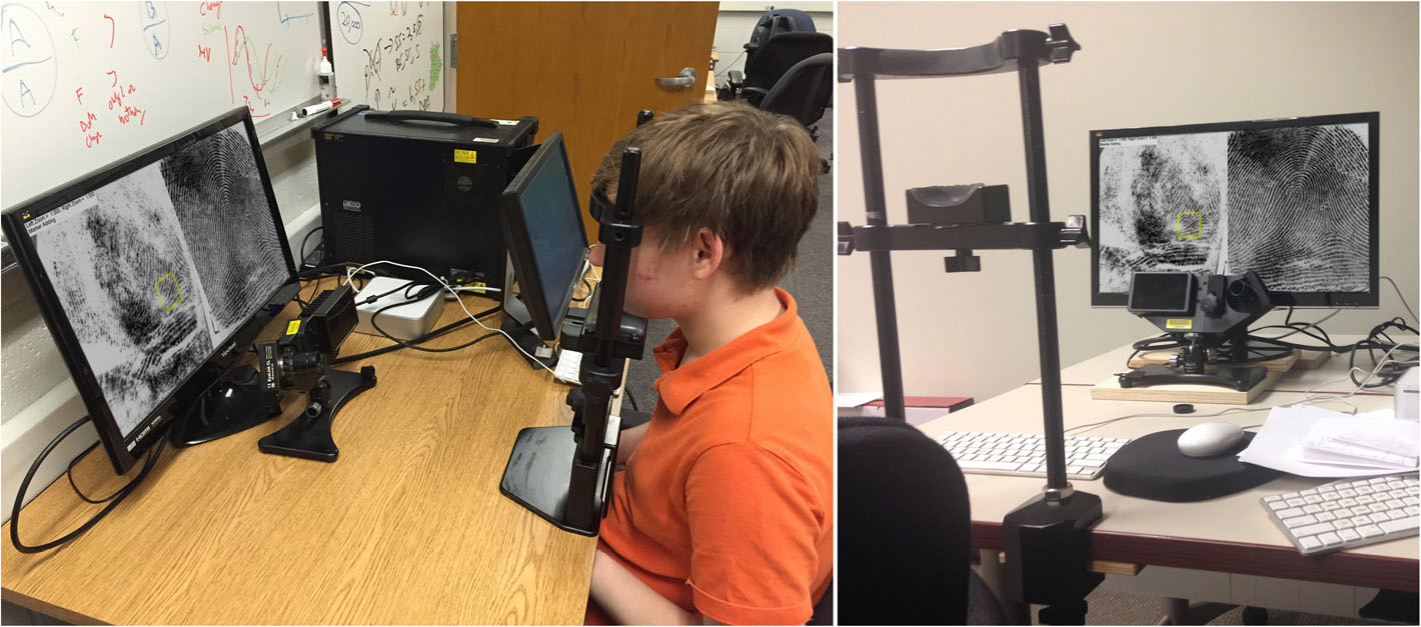
Examples of testing setup. The left image illustrates the main monitor with the eye-tracker below it. The experimenter’s monitor, presentation computer, and eye-tracker computer are to the right of the subject. (Images from actual trials on monitors added for illustration)

Figure 2 shows a typical test setup. Examiners viewed the images on a Viewsonic VX2452mh LCD monitor at 1080p (1920 × 1080) resolution with a 5-ms luminance-change time constant running at 60 Hz. They were positioned using a chinrest 70 cm from the eye to the monitor. At this viewing distance and monitor resolution, there are 50 screen pixels per degree of viewing angle (edge to edge of the monitor was about 38°; average distance between the centers of the left and right images was about 19°).

Presentation code was written in MATLAB (MathWorks, 2012) using functions from the Psychtoolbox for image presentation (Kleiner, Brainard, & Pelli, 2007; Pelli, 1997), and the Eyelink Toolbox for coordination with the eye tracker (Cornelissen, Peters, & Palmer, 2002). The software interface allowed examiners to zoom in and out at the mouse location using keyboard presses, which were also used to pan the image. The two images were presented separately on each half of the monitor and could be zoomed independently. A software tool to mark and link features such as minutiae was available, and was used on 10% of the target-finding trials. Contrast inversion was also available but seldom used for the present task. The default zoom level was 1:1 (one screen pixel to one image pixel); because the screen resolution was 3.7 ppmm and the image resolution was 39.4 ppmm, there was an effective magnification of 11x. The current zoom levels and markup mode were displayed in the upper-left portion of the monitor as shown in Fig. 2; fixations near that textbox were excluded from our analyses. There were no other user-interface elements (e.g., scrollbars, toolbars, buttons) displayed on the screen.

At the start of the trial, the left image was displayed. When the examiner verbally indicated that they were ready to proceed with the Comparison phase of the trial, the experimenter displayed the right image. When the examiner said that they had found the target, the experimenter terminated the trial and triggered the final drift correction dots.

Gaze location (from both eyes) was recorded at 1 kHz using an EyeLink 1000 eye tracker. The EyeLink camera and illuminator were positioned immediately in front of the monitor on the table, and could be adjusted left or right if necessary to eliminate glare on glasses. We generally used 75% illumination within the EyeLink system, but bifocal contacts or thick glasses that tended to block IR illumination sometimes required 100% illumination.

### Calibration

At the start of, and periodically during, the experiment, the experimenter calibrated the eye tracker. Calibration was done within the EyeLink software using 13 calibration dots; gaze locations were tracked using the centroid measurement technique. Calibration was re-attempted as necessary, with a goal of obtaining an average error calculation of 0.5° for both eyes. A value of 0.8° was considered acceptable, although one examiner was only able to achieve 0.94° and 0.97° after seven calibrations, but was still included in the analyses. The calibration error goal of 0.5° corresponds to about ± one ridge (25 screen pixels) at a 1:1 zoom level (68% of fixations in this task were at 1:1). The foveal field of view (area of high acuity) typically corresponds to about 2°, or a diameter of about 100 screen pixels; at a 1:1 zoom level, this is 2.5 mm in image coordinates, or about 4.5 ridges.

In addition to the overall calibration that was managed within the eye tracker, we collected additional data to allow us to correct for gaze drift in post-processing. Each trial began and ended with seven drift correction dots presented in sequence; the examiner was instructed to fixate on each dot of known location for 1.25 s. Post-processing corrected for systematic drift on each trial by comparing gaze points within a threshold distance (2°) from the dot during the 1.25-s interval against the ground truth location. A clustering algorithm based on the mean shift algorithm (Cheng, 1995) was used to determine the largest cluster of at least 20 successive 1-kHz gaze points within the 2° radius; this cluster center was taken as the intended location of gaze for that drift correction dot. By working with raw gaze points, we take advantage of the density of the gaze points (potentially over multiple fixations and micro-fixations) to determine the intent of gaze during the drift correction procedure. Final drift correction was done using QR decomposition (Francis, 1961) that found a second-order polynomial transformation in both dimensions (six total coefficients) that allowed for translation, rotation, and scaling of the gaze points to the ground truth locations. This transformation was then applied to all fixations extracted from the raw gaze stream, as described next.

### Fixation extraction

Each trial consists of a series of eye locations over time: an (x,y,t) path of raw 1-kHz data, which was processed to differentiate saccades and fixations. Because visual information is only coarsely represented during saccades (Ross, Morrone, Goldberg, & Burr, 2001), and because much of the relevant information for fingerprint comparisons is found in smaller details (minutiae), fixations were the fundamental unit of analysis for most of the study results. The raw gaze stream (1-kHz sample data) was partitioned into fixations and saccades using the following approach. We used the Engbert-Mergenthaler saccade detector (Engbert & Mergenthaler, 2006) with a velocity threshold of 8 pixels/s (0.16°/s) and a saccade duration minimum threshold of 9 ms to identify long saccades. As in Port, Trimberger, Hitzeman, Redick, and Beckerman (2016), a saccade required that the eye remain at rest for at least 20 ms within a ± 0.25° X-Y positional window. The Engbert-Mergenthaler saccade detector (Engbert & Mergenthaler, 2006) does a very good job of detecting saccades in which the eye moves more than 1.5° (Port et al., 2016). However, saccades of less than 1.5° tend to include a mixture of what subjectively appear to be even shorter saccades and fixations that are close together. Differentiating saccades from fixations is further complicated by the fact that fingerprint examiners may make very regular, closely spaced, fixations when counting or following ridges. A single saccade detector with a high-velocity threshold may risk grouping together several close fixations into a single fixation (of long duration) that may not accurately represent the detailed behavior. However, a low threshold risks producing many spurious fixations and saccades. Thus, we modified the Engbert-Mergenthaler saccade detector using a variation of the double-threshold algorithm, which is popular in many image-processing applications (e.g., Chen, Sun, Heng, & Xia, 2008) where distance can complement a threshold applied to some other value such as image intensity. Similar approaches have been used for eye-tracking analyses on fingerprint examiners (Busey et al., 2013, 2015; Parada et al., 2015).

**Fig. 3:**
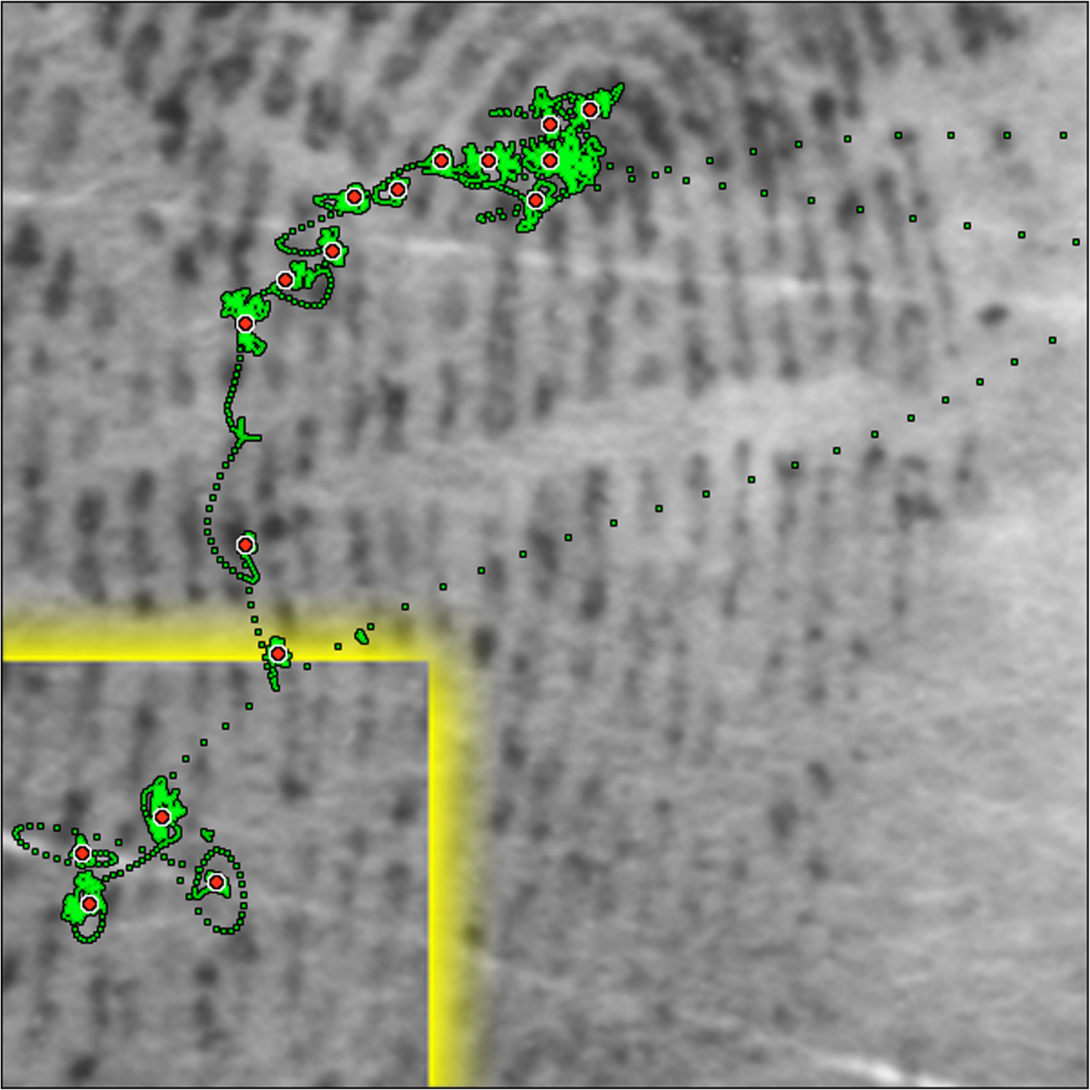
Example of raw 1-kHz gaze data (green) with fixation centroids (red), from a single examiner. This example shows evidence of what appears to be ridge counting and ridge following behavior as a possible means to relate the target region to the core. The relative closeness of the fixations during counting demonstrates the need for a fixation extraction algorithm that uses two velocity thresholds plus additional temporal and spatial constraints as described in the “Fixation extraction” section. A brief pause in the long vertical saccade above the yellow target area was not labeled as a fixation by our extraction algorithm because its duration (50 ms) was less than the 80 ms threshold for a fixation

To apply the double-threshold algorithm, the period between long saccades was further divided into one or several fixations according to the following approach. First, we created a set of candidate fixations by re-applying the Engbert-Mergenthaler saccade detector using a lower-velocity threshold of 3 pixels/s (0.06°/s) and the same saccade duration threshold of 9 ms. The use of a lower threshold has the advantage that it finds more short saccades, at the risk of spuriously introducing extra fixations where the eye does not travel far enough to be considered a true fixation. If two contiguous fixations had centroids within a minimum distance, then these two fixations were merged together under the assumption that the low-velocity threshold may have inadvertently split some true fixations. We selected a minimum distance of 0.35°, which is more conservative than the 1° value recommended as the fixation radius by Blignaut (2009), but tends to preserve closely spaced fixations rather than grouping them into a larger fixation. This approach was necessary because at times the examiners tended to put deliberate fixations in close proximity, such as when they were counting or following individual ridges. Figure 3 illustrates the resulting fixations on an example with spatially close fixations.

Fixations were required to be longer than 80 ms, although Manor and Gordon (2003) argued that a minimum of 100 ms can also be justified. The centroid of the fixation was computed as the median x and y location of all of the raw gaze points that are identified as part of the fixation. The resulting mean fixation duration was 320 ms (median 270 ms; quartiles 198–373 ms). The median saccade duration was 21 ms within an image; median duration for crossing saccades (between the left and right image) was 76 ms. Details in Additional file 1: Appendix SI-4.1.

The above procedures determine fixation centroids projected onto the monitor coordinates. These coordinates were then adjusted by the aforementioned drift correction procedures. Because our software allows for scaling and panning of the images, an additional step was required to project the drift-corrected fixations into the image coordinates using the scaling and panning parameters active during that fixation. Additional information regarding fixation extraction methods used and their accuracy are shown in Additional file 1: Appendix SI-4.2.

### Test administration

Each participant was assigned a sequence of fingerprint comparisons, interspersed with three types of directed tasks. In addition to the find-the-target task that is the focus of this paper, the directed tasks included ridge following and ridge counting; results of the fingerprint comparisons and other directed tasks will be reported in subsequent papers. Testing occurred in June–August 2016 in six locations in Ohio, Indiana, Virginia, Kentucky, and Georgia. Participants were provided with written instructions prior to the test. An experimenter then verbally summarized the instructions and answered any questions. Participants were requested to continue testing for 2 h or until all of the assigned trials were completed; however, participants were permitted to stop early or continue after the 2-h time period.

### Participants

Participation was open to practicing latent print examiners who are currently doing casework or have done casework within the last year. Participants gave informed consent after reviewing a human subject consent form approved by the Federal Bureau of Investigation Institutional Review Board prior to the start of the study. A total of 122 examiners participated: 39% were from federal agencies, 31% state, 22% local, 5% international, and 2% private. Seventy-nine percent were from accredited laboratories. Seventy-six percent had 5 or more years of experience as a latent print examiner; none had less than 1 year. Nineteen percent wore glasses, 29% had contact lenses, and 7% had LASIK. No participants were required by their employers to participate. Participants were assured that their results would remain anonymous; a coding system was used to ensure anonymity during our analyses and in reporting. Usable eye-tracking data for the present task was collected from 117 participants: of the total 122, four participants were tested during an initial phase of data collection in which find-the-target tasks were not assigned, and data from one participant was unusable due to a corrupt file. (See Additional file 1: Appendix SI-2 for further details on participants.)

### Test yield

The 117 participants completed a total of 675 valid trials (two invalid trials were omitted). Each examiner was assigned only one type of task (latent, plain, cropped) from each image set (FT1–FT8); therefore, no examiner was assigned two trials involving the same source finger. Because participants were allowed to stop early or continue, some completed as few as two or as many as eight trials; most participants (87) completed at least two trials of each type (latent, plain, cropped). Image sets FT7 and FT8 were retired early during the course of testing to better use the limited time with each examiner; due to the smaller resulting sample sizes, these two image sets are omitted from analyses aggregated by image pair. From 32 to 39 examiners completed each of the tasks from FT1_Latent_ through FT6_Cropped_ (18 image pairs); only seven to nine examiners completed each of FT7_Latent_ through FT8_Cropped_ (six image pairs). The 675 trials included a total of 53,093 valid fixations; for analyses omitting FT7 and FT8, there were 630 trials with 49,242 valid fixations. (See Additional file 1: Appendix SI-5 for further details regarding omitted data and test yield.)

## Describing eye-gaze behavior in localization

In this section, we describe the behavior of examiners during the localization process, and show the effects of context. We also showcase techniques that we developed to visualize and describe eye-gaze behavior.

### Spatial analyses

**Fig. 4:**
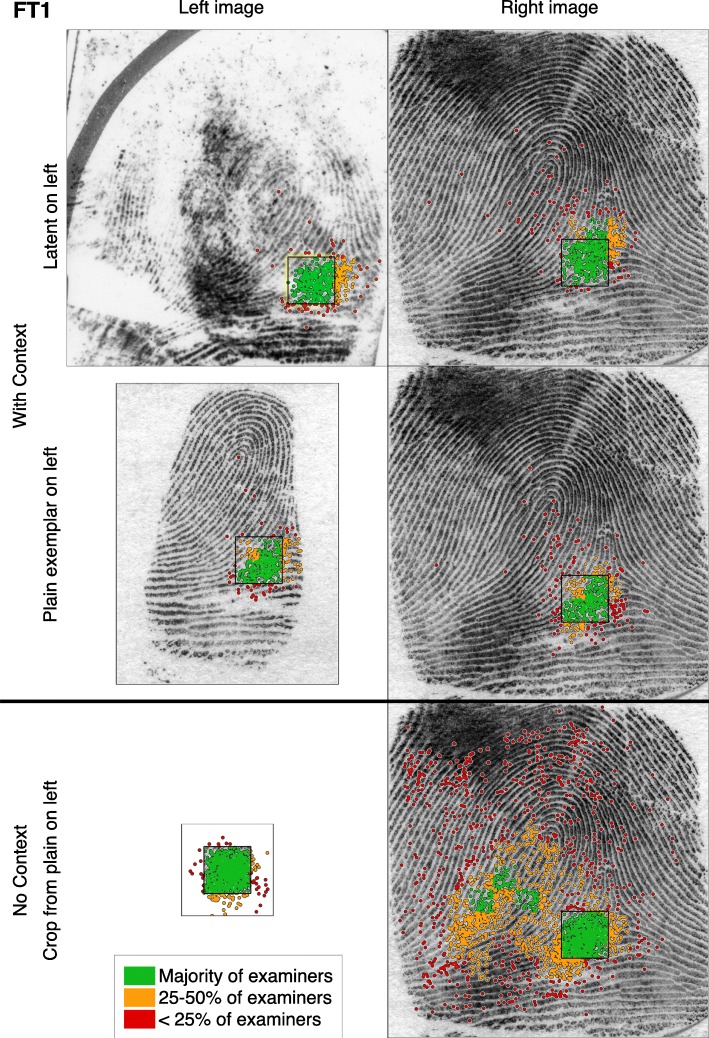
Aggregate of Comparison-phase fixations from all examiners on one image set (FT1). Target area is shown here as a black outline. Color-coding describes the proportion of examiners who had any fixations in each cell. (*N* = 35 examiners (FT1_Latent_), 32 (FT1_Plain_), and 37 (FT1_Cropped_)). FT1 images without fixations are shown in Fig. 1. See Additional file 1: Appendix SI-6.1 for Analysis-phase fixations and other image sets

How does providing visual context affect the spatial distributions of fixations in the right image? Figure 4 depicts the comparison fixations made by 104 examiners on one image set. The three rows of images correspond to the three task types, as determined by the type of the left image. For our spatial analyses, we define an imaginary grid that partitions the image into cells, each one quarter the size of the target area (each cell is 75 × 75 pixels, or approximately 1.5° of visual angle). We define the “popularity” of an area (color coding in Fig. 4) as the proportion of examiners who had any fixations in a given cell. We also use these cells to describe the extent of the area where an individual examiner looked, defining the “area visited” as the number of cells with any fixations from that examiner.

Context had a significant effect on the number and spatial distribution of comparison fixations in the right image. With context (latent and plain tasks), most fixations were in or near the target area (for both left and right images): less than 4% of fixations were farther than one target width (approximately 3° of visual angle) from the target area; examiners needed few fixations far from the target in the right image to get an overall sense (gestalt) of the print. Without context (cropped tasks), examiners looked at much more of the right image, with far more fixations, and with more areas considered by many examiners: 36% of fixations were more than one target width away from the target area. The median area visited by examiners in the right image on cropped tasks was more than triple that of plain tasks, and nearly triple that of latent tasks (as measured by the count of grid cells visited; details in Additional file 1: Appendix SI-6.3).

Most image sets included areas in the right image not adjacent to the target that drew the attention of many or most examiners on the cropped trials. Such “decoy” areas tended to have pattern flow similar to the target area. In Fig. 4, the ridges in the target area converge from the top right to bottom left; such ridge flow is found in two areas in the fingerprint, the left side of the delta (where the target is) and the lower left of the core (“decoy” areas). Pomplun (2006) characterized such decoy areas by calculating the *saccadic selectivity*, and found that observers were more likely to fixate on regions in natural images that are similar to the target along dimensions such as intensity, contrast, orientation, or spatial frequency. In our stimuli, other elements, such as curvature and ridge flow, are also likely guiding saccadic behavior. Additional file 1: Appendix SI-6.1 shows other clear examples of saccadic selectivity, in which the decoy areas attract a large number of fixations in the cropped task. However, the addition of context almost completely eliminates the fixations to decoy areas, as well as much of the scanning and searching behavior seen in cropped trials. This illustrates the powerful constraints provided by context in our task.

**Fig. 5:**
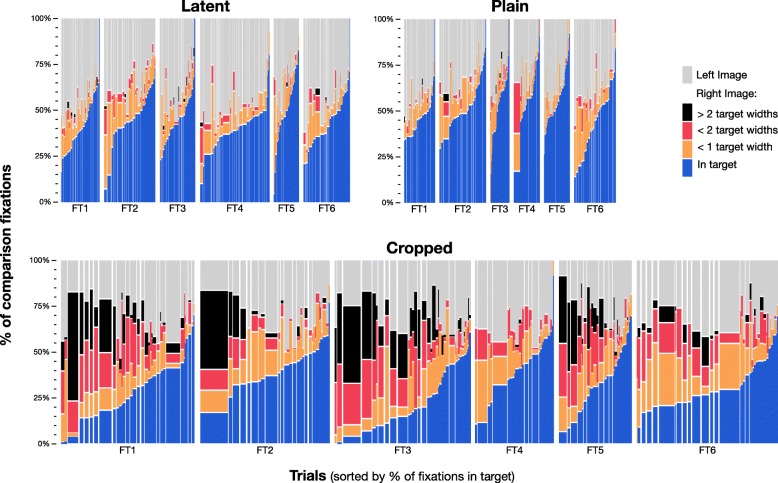
Distance of fixations from target, by task type, image set, and trial. These mosaic plots describe for each trial the proportion of comparison fixations on the left and right images, and by distance from target in right image, for each task type. Each column is one trial, sorted by the percentage of fixations in the target on the right image; column width is proportional to number of fixations in that trial. (*n* = 8956 fixations (Latent), 6171 (Plain), 20,116 (Cropped))

Figure 5 summarizes the spatial data for each trial to show the interexaminer variation in the number and relative location of fixations during the Comparison phase. Some examiners contributed many more fixations than other examiners (wide columns), and examiners differed greatly in the number and proportion of fixations far from the target (red and black), particularly in the right image of cropped trials. For example, two examiners made half of all the fixations more than two target widths away from the target in FT1 cropped (black areas), and, therefore, are disproportionately responsible for the fixations far from the target we see in Fig. 4. In cropped trials, each examiner typically visited only a relatively small proportion of the right image: if we compare the area visited by individual examiners to the total area visited by all examiners in that image, the median area visited was about 15–20% of the total area (details in Additional file 1: Appendix SI-6.2).

### Time to complete

**Fig. 6:**
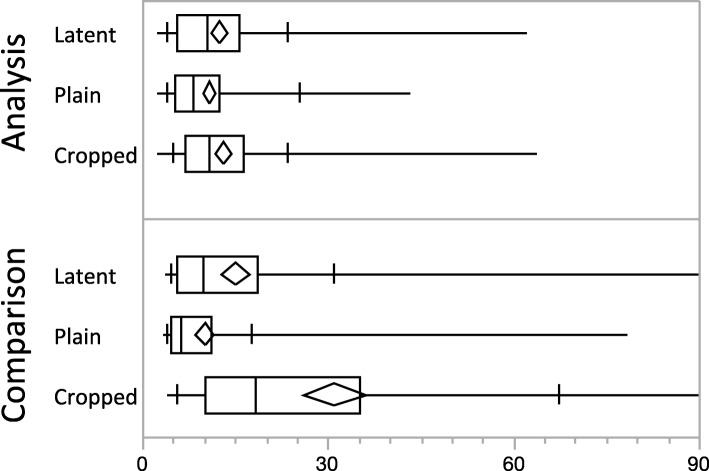
Total Analysis and Comparison times. Analysis medians (seconds): Latent = 10.2; Plain = 8.1; Cropped = 10.7. Comparison medians (seconds): Latent = 9.7; Plain = 6.1; Cropped = 18.2. Crossbars indicate 10% and 90% deciles. Maximum Comparison times (not shown): Latent 152 s (1% of trials > 90 s); Cropped 281 s (7% of trials > 90 s). (*n* = 675 trials)

The total task completion times were approximately log-normally distributed, varying from 6 s to 5 min (inter-quartile range: 14 to 37 s). As shown in Fig. 6, the total time examiners spent on Analysis (studying the first image prior to being shown the second image for comparison) was similar across the three task types (slightly shorter for plain tasks). However, the time spent on Comparison varied substantially by task type: Comparison times were generally much longer for cropped tasks than for latent tasks and plain tasks. Beyond task type, some of the variability in total time spent on trials can be attributed to a learning effect: median Analysis time for first trials was about twice that for later trials; examiners’ first trials of each type typically had longer Comparison times, particularly for cropped trials (details in Additional file 1: Appendix SI-7.2). When asked, some participants confirmed our conjecture that this reflected a realization that detailed analysis was not required for this limited task. The first trial for each examiner was generally associated with larger area visited and more fixations.

We expect that the amount of time spent by each examiner was affected by individual motivational factors such as competitiveness, and their interpretation of the appropriate level of diligence for the task.

### Rapid localization

**Fig. 7:**
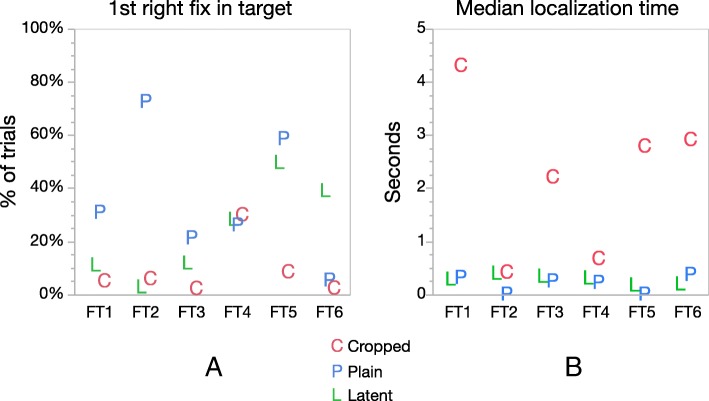
Two measures of rapid localization, by task type and image set. **a** Proportion of trials in which the first fixation in the right image was in the target. **b** Median localization time, measured from the first fixation in the right image to the first fixation in the target area on the right image. See Additional file 1: Appendix SI-8 for distributions of localization times. (*n* = 626 trials; 4 trials had no localizations)

Examiners frequently fixated in the target area of the right image within the first few fixations. We refer to this behavior as “rapid localization.” To quantify this observation, we measured the time from the first fixation in the right image to the first fixation in the target area of the right image. As shown in Fig. 7, for plain or latent tasks, examiners usually localized rapidly: often the first fixation in the right image was in the target area, and in the majority of trials, the examiners looked in the target area within 0.5 s (in the first two fixations). Examiners looked in the target area within the first three right fixations in 78% of latent trials and 91% of plain trials, but only 40% of cropped trials (Additional file 1: Appendix SI-8). For cropped tasks, the localization time varied notably by image set: the majority of examiners first looked in the target area within two to eight fixations.

Some examiners started fixating on the right image 150 ms after it was presented (regardless of task type). In approximately half of trials, examiners first fixated on the right image within 250 ms after it was presented. At approximately 0.5 s, some examiners had already turned their attention back to the left image; after 2 s, examiners in about half of trials had done this, including 15% who had looked right-left-right (details in Additional file 1: Appendix SI-8).

How is localization accomplished so rapidly when context is provided? In plain or latent tasks, the paucity of fixations outside the target area on both the left and right images indicates that examiners either know where to expect the target area to be shown on the screen (even prior to the right image being shown), or acquire coarse information regarding ridge flow from the right image from peripheral vision prior to the first fixation in the right image. Drew et al. (2013) observed such rapid understanding of an image with expert radiologists, noting that experts use nonselective processing that “extracts information from global or statistical information without selecting specific objects.” Greene and Oliva (2009) describe similar rapid image processing using a set of global primitives such as “openness” (for outdoor scenes) or “transience” (for images such as waves or waterfalls) that allow for rapid image classification. Such similar primitives may exist for fingerprints, such that examiners may quickly determine the Henry classification type (e.g., whorls, loops, and arches (Henry, 1900)), orientation, or whether an impression overlaps another print. Within the medical imaging literature, several authors have argued for an initial holistic processing of medical images followed by a more deliberate, slower search and discovery process (Kundel, Nodine, Conant, & Weinstein, 2007; Kundel, Nodine, Krupinski, & Mello-Thoms, 2008; Nodine, Kundel, Lauver, & Toto, 1996). In Kundel et al. (2008), the authors found that participants made a saccade to more than half of all tumors within the first second, which is consistent with the rapid localization seen in Fig. 7.

**Fig. 8:**
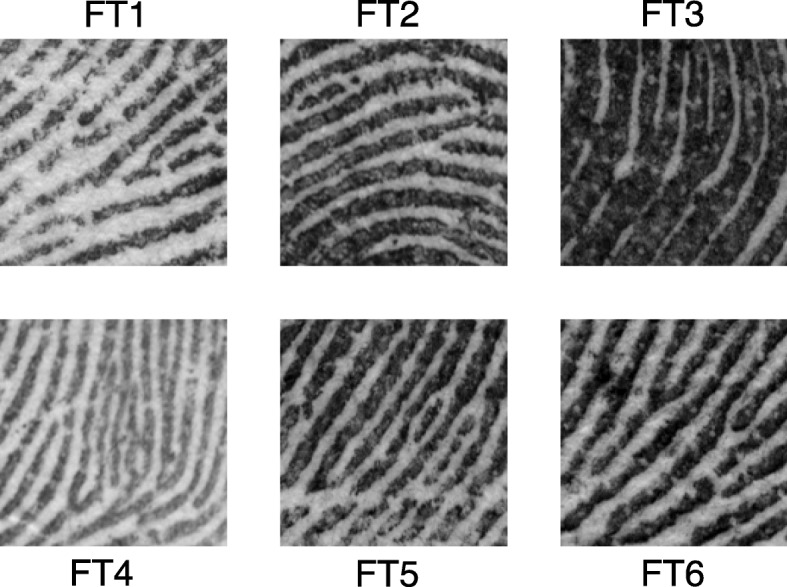
Images used in Cropped tasks from image sets FT1–FT6

For cropped tasks, localization times varied greatly — by image pair and by examiner. We propose that an important factor contributing to this variability is the examiner’s ability to infer contextual information from the cropped images: in sharp contrast to *Where’s Waldo*, information in the cropped images contains clues as to the target location: rapid localization shows that examiners can often utilize this implied context to reduce search time. Figure 8 shows the cropped images from six image sets. The majority of examiners were able to localize FT2 and FT4 in less than 1 s. In general, the direction of ridge flow provides some indication of where the target will be found – examiners were instructed that the images were presented in approximately upright orientation. The direction and curvature of ridge flow in FT2 results from its location just above the core; the convergence of ridges in FT1 and FT6 indicates a location to either side of the lower portion of the loop. In these examples (FT1, FT2, FT6), the features are not highly distinctive, but ridge flow is useful in guiding the search. FT4 is not only in an area of convergence, but contains particularly distinctive features that may have aided examiners in recognizing the correct target.

### Errors finding the target

Our objective was to assess *how* examiners find target groups — not *whether* they would succeed. Given that the participants are practicing latent print examiners, the occurrence of *any* errors on such a simple task is notable. Examiners apparently failed to find the correct target in six of the 675 trials, all in cropped tasks (twice by one participant). In each of these apparent errors, the areas that appear to have misled examiners were the “decoy” areas of the print (areas viewed by many or most other examiners); each of these areas had ridge flow similar to the target. Haste does not appear to have been the cause for four of the six apparent errors, as those trials took longer than the median Comparison time for those image pairs; the two trials that were faster than the median Comparison time were made by the same examiner, who had faster than median Comparison times overall. Miscalibration does not appear to have been a concern for these trials because drift correction was verified prior to and after each trial. Four of the five examiners who made errors (including the examiner with two errors) had less than 5 years of experience; 24% of all participants had less than 5 years of experience. Additional file 1: Appendix SI-9 shows the fixations and images for these six trials.

We bring attention to these localization errors because they suggest how erroneous conclusions in actual comparisons may occur. Although we detected few localization errors, they are still notable: we cannot expect to have rare events in quantity (for comparison, erroneous identification rates are estimated at approximately 0.1% (Ulery et al., 2011)). If, in a real comparison, an examiner selects and compares an incorrect but similar target group, and either does not notice or discounts the correct target group, that could start a path of reasoning that could result in an erroneous identification (if the examiner discounts or does not notice discrepancies) or an erroneous exclusion (by expanding from the wrong location then noting invalid “discrepancies”). Ulery et al. have previously observed (Ulery et al., 2014, 2016) examples of erroneous conclusions where examiners’ markups revealed misassociated features. Given the seriousness (albeit rarity) of erroneous identifications, and a higher than expected erroneous exclusion rate in latent print examination (Pacheco et al., 2014; Ulery et al., 2011), it is important to highlight faulty localization as a behavior that could help explain such erroneous conclusions.

### Timelines

**Fig. 9:**
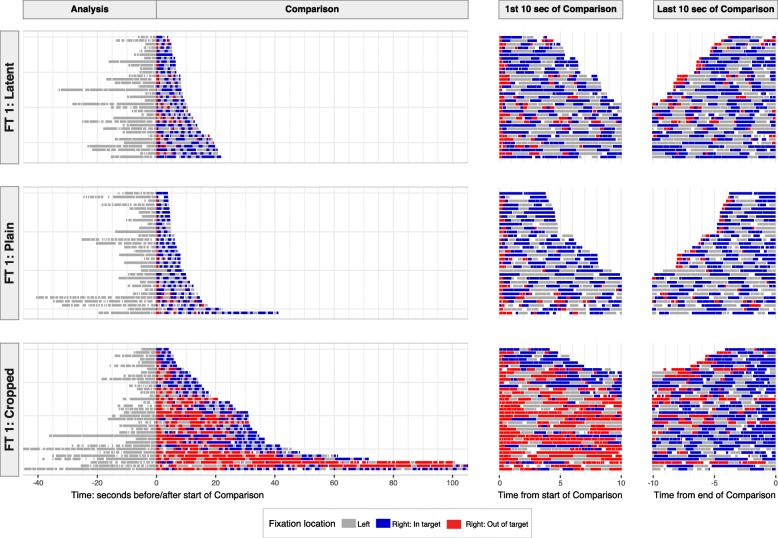
Timeline view of all trials for one image set (FT1). Each row is one trial (i.e., different examiner); trials are sorted by Comparison time. (Left) Analysis and Comparison times are shown relative to the first fixation in the right image. (Right) First and last 10 s of Comparison phase. Each horizontal segment represents one fixation, with gaps shown in white (brief gaps are saccades; longer gaps are blinks or looking away from images). Longest timelines are truncated (longest Analysis times: 53, 64 s; longest Comparison times: 133, 167 s). Timelines for other image sets are in Additional file 1: Appendix SI-10. Left image fixations are not color-coded by location (in or out of the target area) because they are overwhelmingly in the target area (Additional file 1: Appendix SI-6.2)

For a graphic visualization comparing eye-gaze behavior across all trials for a given image pair, we combine spatial and temporal information into a “timeline” view. Figure 9 (and the timelines for other image pairs in Additional file 1: Appendix SI-10) shows several notable patterns:

- There was substantial variation in Analysis times. We found no strong correlation between Analysis and Comparison times (there was a very weak positive correlation; see Additional file 1: Appendix SI-7.1). We might have expected a negative correlation if lengthy analysis were to result in faster target finding or decision-making, or a positive correlation if individual examiners were consistently slower or faster in both phases. The weak observed correlation could reflect both opposing influences
- Examiners generally spent more Comparison time in the right image than the left image: median 82% of Comparison time was in the right image for cropped tasks, 76% for plain tasks and 67% for latent tasks — presumably because the left image in latent tasks is poorer quality and, therefore, requires a greater proportion of Comparison time. However, the repeated returns to the left image suggests that subjects are reluctant to trust short-term memory, or the contents of short-term memory are fading. Thus, they use the physical presence of the left image to reduce the demands on short-term memory (Ballard et al., 1995). During Comparison, examiners frequently switched back and forth between the left and right images. Examiners generally spent 1 to 3 s on an image before looking at the other image, except for the right image in cropped trials, in which they generally spent 2 to 6 s (inter-quartile ranges; details in Additional file 1: Appendix SI-13.3)
- In cropped tasks (but not latent or plain tasks) many examiners spent an extended period outside the target prior to a final period in the target area. Much of the variability in the Comparison time of cropped trials can be accounted for as differences in the amount of time spent outside the target. The total amount of time spent in the target area prior to announcing that the target was found was similar across trials and tasks: in plain and latent trials examiners generally fixated in the target area in the right image for 3 to 6 s (inter-quartile range) or 4 to 11 s for cropped trials (details in Additional file 1: Appendix SI-11).

## Characterizing eye-gaze behavior in localization

The spatial plots and timelines are powerful visualization techniques for exploring this complex dataset; they can be used as qualitative descriptions and to develop hypotheses and general explanations. However, in order to measure the behavioral patterns we observed, we need additional tools to characterize eye-gaze behavior. In this section, we partition trials into subphases and define behavioral metrics that are associated with these subphases.

### Summarizing trials in terms of subphases

The timelines reveal notable differences by task type and between trials in terms of behavior with respect to the target in the right image: *How long does the examiner take to find the target? After the examiner finds the target, how long does the examiner keep looking elsewhere? How long does the examiner remain looking in the target at the end of Comparison?* In order to measure each of these, we partition the Comparison-phase timelines into three corresponding “subphases”: we define Subphase A as the period before the examiner finds the target, Subphase C as the period after the examiner stops considering any areas outside the target, and Subphase B as the in-between period (detailed in Table 1). Subphase A is generally brief in latent and plain tasks but often lengthy in cropped tasks, Subphase B is only present in some trials (most prevalent in cropped tasks), and Subphase C is present in almost all tasks. See Additional file 1: Appendix SI-12 for additional discussion of partitioning timelines into subphases.

**Table 1:** Definitions of subphases of trials

Subphase	Definition	Example
A	A period at the *start* of Comparison prior to the examiner first looking at the target area, in which all fixations in the right image are outside the target area. Because Subphase A was often very brief, we use “A_L_” and “A_S_” to indicate whether the subphase was longer or shorter than 1 s	In Fig. 9, for trials in which the first right fixation is red (out of the target area), Subphase A is the period prior to the first continuous 0.5 s of blue (in)
B	Any period of time not in Subphase A or C	In Fig. 9, Subphase B is a period alternating between blue and red (after initial red and final blue periods)
C	A period at the *end* of Comparison when the examiner is only looking at the target area, in which all fixations in the right image are in the target area in the right image	In Fig. 9, for trials in which the last right fixation is blue (in the target area), Subphase C is the period after the last 0.5 s of red (out), until the end of the trial; for trials that have no red, the entire Comparison phase is considered Subphase C

We can use the presence or absence of subphases to classify entire trials, which allows us to summarize the differences among trials. For example, a trial with all three subphases present is labeled “*A*_*L*_*BC*” or “*A*_*S*_*BC*,” and a trial with only the C Subphase is labeled “*C.*” The presence or absence of subphases results in ten possible combinations of subphases, as shown in Table 2. In latent and plain trials, the examiner often went immediately to the target and stayed there (*C*); in a majority of latent and plain trials, the examiner localized the target with at most a brief orientation, then stayed there (*C* or *A*_*S*_*C*). Cropped trials differ most notably from latent or plain trials in that examiners generally did not find the target quickly (*A*_*L*_*C* or *A*_*L*_*BC*). Median Comparison times for *C* and *A*_*S*_*C* trials are similar across all task types (5.0–8.1 s). Regardless of task type, long Comparison times are usually due to lengthy Subphases A and/or B. Other authors have suggested two separable stages in naturalistic search tasks such as chest x-ray and mammogram inspections: a rapid holistic processing stage that guides an initial fixation and a slower, more deliberate process that involves scanning and discovery (Donovan & Litchfield, 2013; Kundel et al., 2007; Nodine, Mello-Thoms, Kundel, & Weinstein, 2002). A rapid holistic search would appear as a very brief Subphase A.

**Table 2:** Categorization of trials in terms of presence or absence of subphases, with proportions of trials and median comparison times. Rows with similar subphase combinations are grouped. Median times based on three or fewer trials are shown in italics. (*n* = 675 trials)

Subphase combination		% of trials			Median comparison time (secs)		
	Description	Latent	Plain	Cropped	Latent	Plain	Cropped
C	Only looked in target	12.3%	25.4%	2.2%	5.3	5.1	6.1
A_S_C	Quickly found target, then only looked in target	37.9%	41.2%	16.2%	8.1	5.0	7.0
A_L_C	Took time to find target, then only looked in target	23.3%	13.6%	42.1%	10.9	8.9	15.7
BC	Started in target, continued looking elsewhere, then only looked in target	2.7%	4.0%	0.9%	18.5	12.7	*26.8*
A_S_BC	Quickly found target, continued looking elsewhere, then only looked in target	8.7%	7.9%	10.1%	22.0	10.8	31.8
A_L_BC	Took time to find target, continued looking elsewhere, then only looked in target	10.5%	5.3%	24.1%	24.1	16.3	43.5
B	Started in target, but did not end up there	0.5%	0.4%	0.0%	*4.8*	*4.0*	*–*
A_S_B	Quickly found target, but did not end up there	0.9%	1.3%	0.0%	*7.2*	*6.6*	*–*
A_L_B	Took time to find target, but did not end up there	1.8%	0.4%	2.2%	12.4	*78.2*	26.7
A_s_	Quickly completed but never found target	0.0%	0.0%	0.0%	*–*	*–*	*–*
A_L_	Took time, but never found target	1.4%	0.4%	2.2%	*7.8*	*7.2*	21.0

Although here Subphases A and C are defined in terms of the start and end of an isolated find-the-target task, full fingerprint comparisons will generally consist of multiple localization events in series (e.g., ABCACABCAC), and, therefore, Subphase C would often be followed by another Subphase A.

### Characterizing transitory overt behaviors

Our decomposition of trials into subphases is only possible because in this test we have ground-truth knowledge of where the target locations are, and a narrowly defined task with a single target. In real-world comparisons, ground-truth knowledge of predefined targets would not be available, and subphases would be more difficult to determine. It is, therefore, desirable to be able to describe what a fingerprint examiner is doing at a given time in a comparison; such “overt behaviors” describe transitory attributes of eye behavior, and are measured over individual fixations or series of consecutive fixations. Because measures of overt behavior do not require ground-truth knowledge (i.e., make no reference to the target locations), they may be more generally applicable to real-world fingerprint comparisons. We define four metrics of overt behavior (italicized):

- *Speed3* measures the speed of eye movement within an image in image pixels per second. For each fixation, *Speed3* is measured over a series of up to ± three fixations, as the sum of inter-fixation distances, divided by the time from the start of the first fixation to the end of the last fixation in the series. Fixations that are near left-right transitions will have fewer than ± three fixations in the series
- *PercentOfFixesInCell* measures the percentage of fixations in a trial that are located in each cell (as defined in the “Spatial analyses” section). For each fixation, *PercentOfFixesInCell* is calculated for the cell in which the fixation is located. This local spatial density measure may reflect an examiner’s level of interest in an area
- We use “image visit” to refer to a consecutive series of fixations in an image (left or right), bounded by the transitions into and out of that image.
  - ○ *TimeInImage* is the duration of time spent in each image visit, from the start of the first to the end of the last fixation in that image
  - ○ *DetailedBackAndForth* is a count of image visits in which the examiner returns to the same small area in consecutive image visits in that image. The examiner is considered to have returned to the same small area if the distance between the centers of mass of consecutive image visits in the same image is within 88 pixels (approximately four ridges), and the maximum distance between any two fixations in each image visit is no more than twice that distance

There are strong relations between these metrics for overt behaviors and subphases, which we will use as a basis for inferring cognitive states in the next section. The speed of eye movement is generally slower in Subphase C, faster in A_L_, and much faster in A_S_ (medians: A_S_ = 11.9 ridges/s; A_L_ = 10.3, B = 7.6, C = 4.5). During Comparison, the median *TimeInImage* was about 3 s for the right image in cropped tasks; 1–2 s otherwise. Subphases C and A_S_ have shorter *TimeInImage* than A_L_ or B (medians: Subphase A_S_ = 1.7 s, A_L_ = 3.2, B = 3.8, C = 1.9). *DetailedBackAndForth* is nonzero in a quarter of the fixations in Subphases B and C, but is almost always zero in Subphase A. *PercentOfFixesInCell* was notably higher for cells in the target area (and, therefore, for Subphase C). Details are in Additional file 1: Appendix SI-13.

## Inferring cognitive states

Having developed a set of measures that characterize performance in our target localization task, we now turn to the goal of inferring the underlying cognitive states that generated the observable gaze behavior. During an actual comparison, localization decisions may occur multiple times (unlike in this directed task); in the more naturalistic setting of actual comparisons we would like to separate the continuous stream of fixations into discrete cognitive states. However, one challenge is that the subphases described in the “Summarizing trials in terms of subphases” section are defined relative to the known target location, which will not be available in casework-like comparisons. To address this challenge, we explore the relation between the metrics developed in the “Characterizing transitory overt behaviors” section and the subphases to determine how accurately we can predict the underlying subphase given a set of observable metrics *that do not depend on knowing the target location*. We develop this relation so that we can address this question in casework-like comparisons: if an examiner has one or more target groups in memory and is searching for a correspondence between two impressions, can we determine when the examiner believes that they have found a correspondence?

### Model specification

A first step in this modeling process is to determine a measure of cognitive state. We viewed talk-aloud protocols as too intrusive and not representative of casework-like settings. Instead, subphases serve as proxies for an examiners’ cognitive state or intent. Subphase A (especially A_S_) usually has fast eye movement with fixations far apart and relatively little back-and-forth movement, indicating a “where is it?” period of looking for potential locations, roughly corresponding to Kundel’s “Scanning” (Kundel et al., 1978). Subphase C generally has slow eye movement and fixations close together, often with detailed back-and-forth to the same location, indicating detailed work, consistent with an “am I sure?” period of deciding whether it is the correct target, roughly corresponding to Kundel’s “Decision.” Subphase B appears to be an “Is this it?” period, alternating between A_L_-like behavior and C-like behavior.

To provide a link between overt behavior and underlying cognitive states, we developed a set of logistic regression models to assess the association of behaviors with subphases. We are particularly interested in predicting Subphase C (indicating that an observer has entered a decision or correspondence portion of the target group comparison process) and Subphase A (indicating scanning for a target group). We do not explicitly model Subphase B because its relation to any underlying cognitive state(s) is ambiguous, and, therefore, Subphase B is less theoretically interesting.

Each right-image fixation is labeled with a subphase according to the definitions in Table 1. We evaluate several logistic regression models to provide an indication of the strength of association between the measures introduced in the “Characterizing transitory overt behaviors” section and underlying cognitive states, as estimated by subphases. We predict Subphases A and C in separate binary models: we model A vs. all other fixations (B and C), and separately model C vs. all other fixations (A and B). By modeling in this way, we can identify the strength of the relation between our gaze-behavior measures and what we interpret as the Scanning and Deciding cognitive states.

### Model performance

Table 3 summarizes the effectiveness of the various metrics at predicting subphases on tasks with and without context provided. The different measures vary in their ability to predict different subphases, with *Speed3* and *PercentOfFixesInCell* as the two strongest predictors (generally achieving area under the curve values (AUCs) greater than 0.7, and often greater than 0.8). Classification accuracy improves when multiple measures are combined, but the limited improvement indicates that the various metrics are not highly complementary and, therefore, these behaviors are interrelated. Note that although *PercentOfFixesInCell* was indicative of subphase on this task, it might be less effective in full fingerprint comparisons with multiple target groups because the fixations would be less concentrated.

**Table 3:** Performance of logistic regression models predicting the subphase or location of fixations (*n* = 22,747 Comparison-phase fixations on the right image from 675 trials). Model performance is compared using area under the (receiver operating characteristic) curve (AUC). No cross-terms were used in these models: each model had one degree of freedom for each predictor term

Model	Cropped 13,770 fixations		Latent and Plain 8794 fixations	
	Subphase A	Subphase C	Subphase A	Subphase C
*Speed3*	0.73	0.81	0.80	0.69
*PercentOfFixesInCell*	0.67	0.84	0.74	0.73
*TimeInImage*	0.46	0.75	0.60	0.52
*DetailedBackAndForth*	0.63	0.68	0.65	0.57
*Speed3* + *PercentOfFixesInCell*	0.74	0.87	0.83	0.76
*Speed3* + *TimeInImage*	0.72	0.86	0.79	0.70
*Speed3* + *DetailedBackAndForth*	0.74	0.83	0.81	0.69
*TimeInImage* + *DetailedBackAndForth*	0.66	0.79	0.72	0.56
*Speed3* + *TimeInImage* + *DetailedBackAndForth*	0.74	0.86	0.81	0.70
*Speed3* + *PercentOfFixesInCell* + *TimeInImage* + *DetailedBackAndForth*	0.78	0.89	0.84	0.76

The logistic regression results show that the subphases are associated with basic differences in eye behavior and our metrics are effective in detecting these differences. More broadly, we believe that the association between real-world gaze behaviors (as measured by these metrics) and the underlying cognitive states (as approximated by the subphases) is robust enough to be useful when analyzing gaze behavior from casework-like comparisons. In such analyses, models developed from the associations shown here can be used as potential indicators of Scanning and Deciding cognitive states, providing a heretofore unavailable means of assessing how fingerprint examiners conduct comparisons and make determinations.

## Discussion and conclusions

The purpose of this study is to describe and characterize the eye behavior of experts in a localization task, which we intend to serve as a basis for the deconstruction of the overall fingerprint comparison process. In particular, we develop methods for evaluating eye-gaze behavior during localization, describe the behaviors observed, and develop models that link the observable eye-gaze behavior to underlying cognitive states, providing an indication of when an observer believes that they have found a corresponding location for a target region.

There were significant differences in the eye behavior of examiners for tasks with or without visual context. The absence of visual context (cropped tasks) was generally associated with longer Comparison times, and larger areas of the image viewed. For the same image pair and task (particularly in cropped tasks), there was substantial variability among different trials in terms of which areas of the image were viewed, and in Analysis and Comparison times. Trials generally had relatively little variation in the amount of time spent looking in the target area: most of the variability in Comparison time can be attributed to differences in the time spent looking in nontarget areas. When visual context was provided, examiners usually located the correct target area within 1 s. When context was not explicitly provided, examiners appeared to infer information about the context from characteristics in the cropped target area, such as ridge curvature and patterns of convergence. Several of the fingerprints included areas with pattern flow similar to the target (“decoy” areas) that attracted the attention/gaze of a majority of examiners on cropped trials. The large number of fixations to these decoy areas in the cropped task is consistent with visual feature guidance based on similarity along basic visual dimensions (Pomplun, 2006), although the relevant dimensions for fingerprints may be different than those for natural images.

Beyond the information provided by explicit spatial context (i.e., the position of the target area in the left impression), there may be more experience-based contextual information that guides eye movements, even in the cropped images that do not provide explicit content. Examiners may acquire global shape information from expertise-based expectations of fingerprint structure and peripheral mechanisms that provide overall shape of ridge flow, both of which are likely to be much more effective when context is provided. This global shape information may be used to localize rapidly and to avoid decoy areas. These same peripheral mechanisms likely support the re-acquisition of a potentially corresponding location when the examiner must make a saccade back to the left image.

In several trials, examiners failed to locate the correct target area: these examiners located incorrect but similar “decoy” target groups, and declared that they found the target. Although we observed few errors, it is notable that practicing latent print examiners made *any* such errors. We bring attention to these errors because they suggest how erroneous conclusions in actual comparisons may occur: erroneous identifications, erroneous exclusions, or inappropriate inconclusives could all result from incorrect localizations.

To infer the cognitive state of fingerprint examiners, we developed a method to partition the localization process into subphases; although the subphases are defined here based on ground-truth knowledge of the correct target locations, the subphases act as proxies for these cognitive states. We then demonstrated an ability to predict these cognitive states directly from eye-tracking data (not using the ground-truth knowledge), indicating potential for use in flagging when an examiner appears to have found a corresponding location in conventional fingerprint comparisons. These methods provide a tool for determining the extent to which incorrect correspondences of target groups can explain differences in examiners’ conclusions, particularly erroneous conclusions. The analytical methods developed here to assess this simple but critical process are intended as a stepping stone for use in future analyses of examiner gaze behavior in the more complex processes involved in fingerprint comparisons, which generally can be expected to consist of multiple localization events in series. In such analyses, models developed from the associations shown here can be used as potential indicators of Scanning and Deciding cognitive states, providing a heretofore unavailable means of assessing how fingerprint examiners conduct comparisons and make determinations.

Understanding how and why expert latent print examiners make errors and reach different conclusions requires detailed evaluation of fingerprint examiners. This study is a first step in that process: to describe and characterize the eye behavior of experts in a localization task, serving as a basis for the deconstruction of the overall fingerprint comparison process. We intend for the findings and methods discussed here to be used as tools in the evaluation of full fingerprint comparisons — using eye-gaze data collected in the same sitting as these localization trials. When evaluating eye-gaze behavior in full fingerprint comparisons the models developed here can be used to isolate localization behavior, indicating the areas that examiner considers to be corresponding between the prints being compared. Differences among examiners in what they consider correspondences may be used to explain differences in fingerprint comparison conclusions. Lack of localization behavior is also of interest: if an examiner concludes a comparison with little or no localization behavior, the resulting decision is holistic (as might be expected when excluding fingerprints or unrelated patterns). In ongoing and future work, we will use the localization behavior of individual examiners to understand the extent to which errors and differences in conclusions can be explained by differences in behavior.

## Additional files

Additional file 1: Supporting Information Appendices. (PDF 40000 kb)

Additional file 2: Tables of Fixation Data. (XLSX 7142 kb)

## Footnotes

1 See Additional file 1: Appendix SI-1 for glossary of fingerprint-specific terminology.
