# Supplementary material for: Gaze behavior and cognitive states during fingerprint target group localization
Source: Cogn Res Princ Implic. 2019 Apr 5;4:12. doi: 10.1186/s41235-019-0160-9 (PMC6450991; doi:10.1186/s41235-019-0160-9)
Supplement: Supplementary file 1 — Supporting Information Appendices. (PDF 40000 kb) [file 41235_2019_160_MOESM1_ESM.pdf]

Gaze behavior and cognitive states during fingerprint target group localization

Supporting Information Appendices

Contents

Appendix SI-1 Glossary and abbreviations ... 1
Appendix SI-2 Participants ... 2
Appendix SI-3 Fingerprint Images ... 4
Appendix SI-4 Fixation extraction ... 6
Appendix SI-4.1 Fixation and saccade durations ... 6
Appendix SI-4.2 Fixation extraction details ... 7
Appendix SI-5 Test yield and omitted data ... 7
Appendix SI-5.1 Number of fixations ... 7
Appendix SI-6 Spatial analyses ... 8
Appendix SI-6.1 Fingerprint images with fixations ... 8
Appendix SI-6.2 Distance from target ... 14
Appendix SI-6.3 Proportion of image areas visited ... 15
Appendix SI-6.4 Distance to core ... 17
Appendix SI-7 Time to complete ... 19
Appendix SI-7.1 Overall phase and trial duration ... 19
Appendix SI-7.2 Effect of trial number on Analysis and Comparison durations ... 19
Appendix SI-7.3 Relations between Analysis and Comparison times ... 21
Appendix SI-7.4 Factors accounting for variability in Analysis and Comparison times ... 21
Appendix SI-8 Rapid localization ... 21
Appendix SI-9 Errors finding the target ... 23
Appendix SI-10 Timelines ... 26
Appendix SI-11 Total time in the target ... 29
Appendix SI-12 Subphases ... 30
Appendix SI-13 Overt behavior metrics ... 31
Appendix SI-13.1 Speed3 metric ... 31
Appendix SI-13.2 PercentOfFixesInCell metric ... 32
Appendix SI-13.3 Image visits and TimeInImage metric ... 33
Appendix SI-13.4 DetailedBackAndForth metric ... 34

Appendix SI-1 Glossary and abbreviations

This section defines terms and acronyms as they are used in this paper.

**ACE-V**	The prevailing method for latent print examination: Analysis, Comparison, Evaluation, Verification.
**Analysis phase**	The first phase of the ACE-V method. In this test, the examiner annotated the latent and made a value determination before seeing the exemplar print.
**Comparison phase (Comparison/Evaluation phase)**	The second and third phases of the ACE-V method. In this test, there was no procedural demarcation between the Comparison and Evaluation phases of the ACE-V method; hence, this refers to the single combined phase during which both images were presented side-by-side. For brevity, in this report we use “Comparison” to refer to the Comparison/Evaluation phase.
**Exemplar**	A fingerprint from a known source, intentionally recorded.
**Fixation**	A period of relative stability of the eye gaze during which the majority of visual processing occurs.
**FT**	Find the Target set — in this study, the eight sets of images were numbered FT1 through FT8
**kHz**	Kilohertz

**Latent (or latent print)**	A friction ridge impression (or an image of a friction ridge impression) from an unknown source. Outside of North America, an impression from an unknown source (latent) is often described as a "mark" or "trace," and "print" is used to refer to known impressions (exemplars). In this study, one of the three task types showed a latent print as the left image.
**Minutia**	An event along the path of a single friction ridge, either a bifurcation or ridge ending. Examiners were instructed to mark features such as scars, dots, incipient ridges, creases and linear discontinuities, ridge edge features, or pores as "other" features, not as minutiae. In this study, examiners did not differentiate between bifurcations and ending ridges.
**ms**	Milliseconds
**Plain impression**	An exemplar resulting from the touching of the finger to paper or platen of a livescan device without any rolling motion. (also "flat impression") In this study, one of the three task types showed a plain impression as the left image, and another showed an image cropped from a plain impression.
**ppmm**	Pixels per millimeter
**Rolled impression**	An exemplar collected by rolling the finger across the livescan platen (or paper) from nail to nail. In this study, the right image was always a rolled impression.
**Saccade**	A rapid movement of the eyes during which relatively little visual processing occurs.
**Source**	An area of friction ridge skin used to create an impression. Two impressions are said to be from the "same source" when they have in common a region of overlapping friction ridge skin. The samples in this study were known to be from the same source.
**Target group**	A distinctive group of ridge features (and their relationships) that can be recognized *
**Task type**	The type of find-the-target task, based on the type of the left image (latent, plain, cropped)
**Trial**	In this study, an assignment of one image pair to one examiner.

Appendix SI-2 Participants

Participation was open to practicing latent print examiners who are currently doing casework or have done casework within the last year. Participants gave informed consent after reviewing a human subject consent form approved by the Federal Bureau of Investigation Institutional Review Board prior to the start of the study. Participants were assured that their results would remain anonymous; a coding system was used to ensure anonymity during our analyses and in reporting. 122 examiners participated in the broader study, 117 of whom completed the localization portion of the data collection.

Each participant completed a background survey, summarized here.

**1. Current Employment**		
• U.S. Federal government	48	39%
• U.S. Local government	27	22%
• U.S. Private sector (non-government)	3	2%
• U.S. State government	38	31%
• Non-U.S.	6	5%
**2. Has your agency received accreditation in latent prints?**		
• Yes (for example, by ASCLD/LAB, FQS, or ISO/IEC 17025)	96	79%
• No	26	21%
• Don't know	0	0%
**3. Total number of years employed as a latent examiner**		
• Less than 1 year	0	0%
• 1-4 years	29	24%
• 5-14 years	78	64%
• 15 or more years	15	12%
**4. Type of latent training received**		
• Formal program of instruction for 1 year or more	92	75%

• Formal program of instruction for 6 months to 1 year	26	21%
• Limited formal training (courses, workshops) for less than 6 months	1	1%
• Other	3	2%
**5. Are you certified as a latent print examiner? (Check all that apply)**		
• International Association for Identification (IAI) Certified Latent Print Examiner (CLPE)	51	42%
• Certified or qualified as a latent print examiner by a current or previous employer	59	48%
• National certification (non-US only)	2	2%
• Other certification	2	2%
• No certification	29	24%
(Totals add to over 100%. 19 selected both of the first two options.)		
**6. Are you currently conducting latent examinations on a regular basis (at least weekly over an extended period?)**		
• Yes	111	91%
• No, but I have previously conducted latent examinations on a regular basis	11	9%
• No, I have never conducted latent examinations on a regular basis	0	0%
**7. What percentage of time have you spent over the last year doing latent comparisons?**		
• None: I am not performing comparisons	1	1%
• Less than 10%	7	6%
• 10-25%	7	6%
• 25-50%	11	9%
• 50-75%	44	36%
• 75-100%	52	43%
**8. Does your country or agency have a minimum number of minutiae required to make an individualization (or identification) decision? (e.g. a 12-point standard)**		
• No	110	90%
• Yes	12	10%
(Note: 11 of the 12 examiners who answered Yes were employed in US state or local agencies.)		
**9. Did you use glasses or contact lenses when you did the test?**		
• Contact lenses: Astigmatism correction	7	6%
• Contact lenses: Conventional	26	21%
• Contact lenses: Astigmatism correction AND Bifocal or multifocal	2	2%
• Eyeglasses: Astigmatism correction	6	5%
• Eyeglasses: Astigmatism correction AND Bifocal or multifocal	1	1%
• Eyeglasses: Bifocal or multifocal	2	2%
• Eyeglasses: Conventional	13	11%
• LASIK	9	7%
• None of the above	47	39%
• (No response)	9	7%

* National Institute of Justice (2011) *Fingerprint Sourcebook*. (https://www.ncjrs.gov/pdffiles1/nij/225320.pdf)

Appendix SI-3 Fingerprint Images

This section provides information supporting Section 2.1 in the main document.

Fig. S1 through Fig. S3 show image sets FT3 through FT8 (image sets FT1 and FT2 are shown in Figure 1). In each trial, one of the left images (Latent, Plain, or Cropped) was paired with the right image. The yellow squares indicating the target areas were in the images shown to the participants. Target area is 150x150 pixels (at 1000 pixels per inch). Latent images (left column) are slightly cropped for publication.

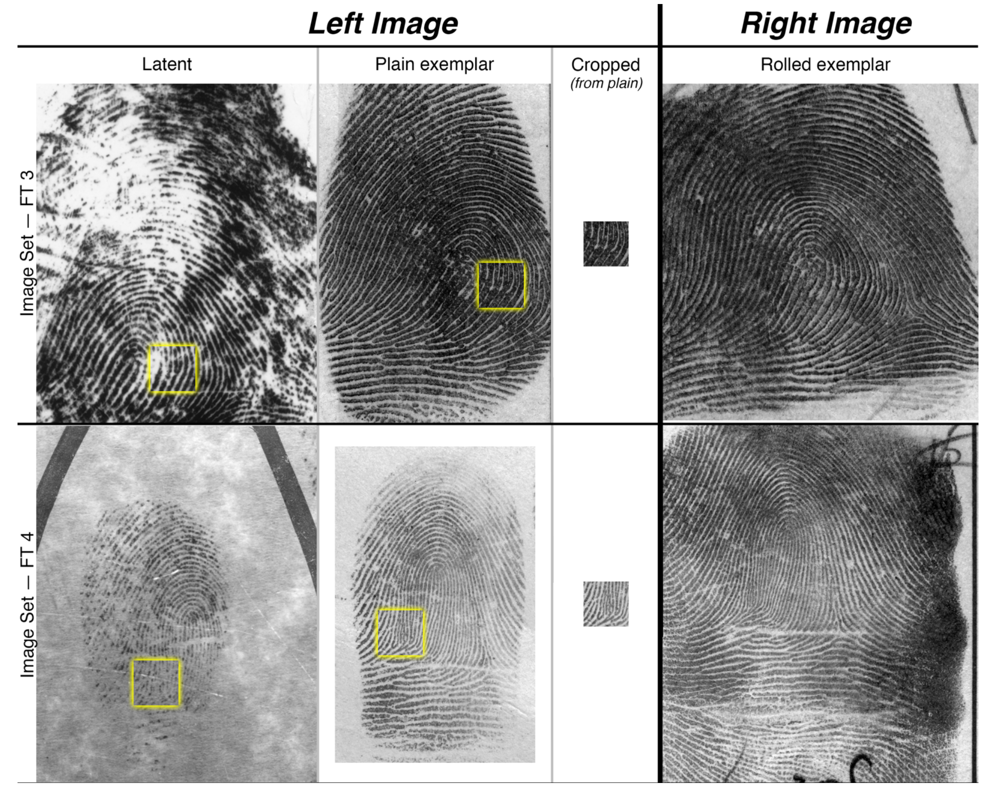

Fig. S1: Image sets FT3-FT4.

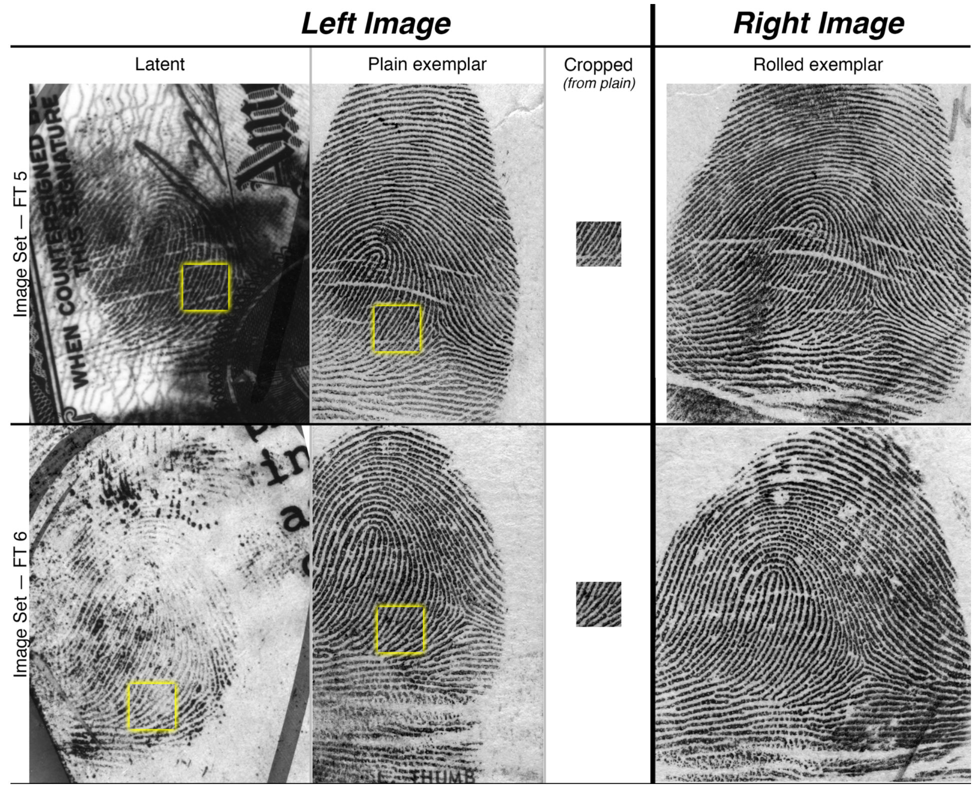

Fig. S2: Image sets FT5-FT6.

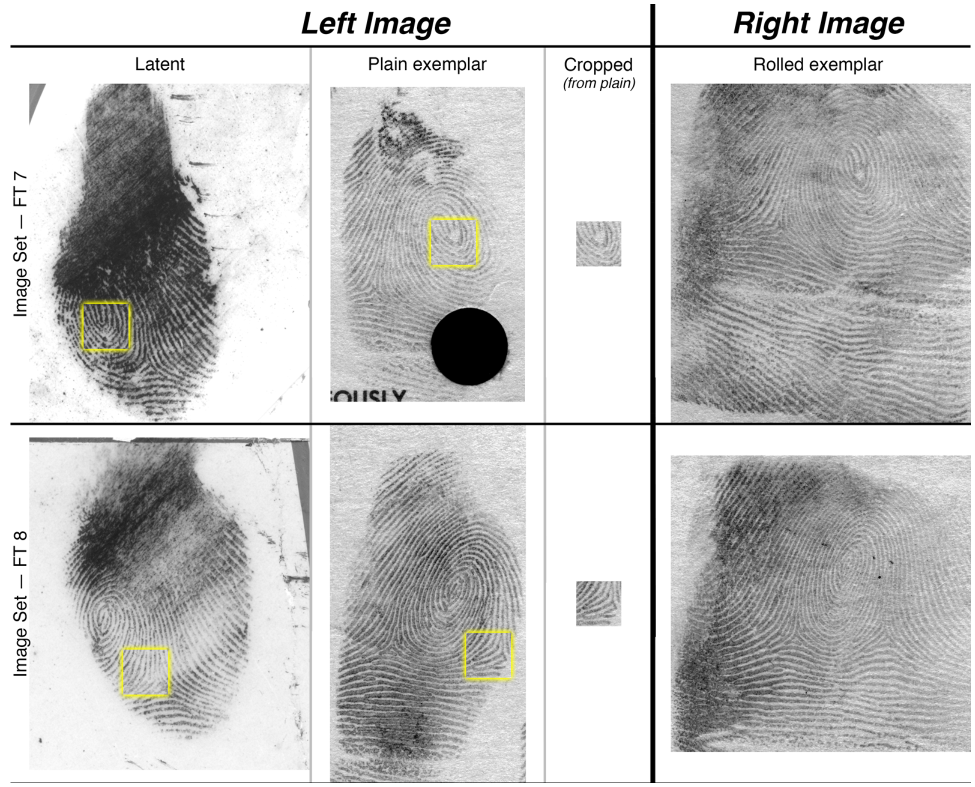

Fig. S3: Image sets FT7- FT8.

Appendix SI-4 Fixation extraction

This section provides information supporting Section 2.4 in the main document.

Appendix SI-4.1 Fixation and saccade durations

Fig. S4 shows the distribution of fixation durations. The minimum fixation duration is defined by the fixation extraction algorithm at 0.08 seconds (described in Section 2.4). Very long fixation durations are affected by the fixation clustering algorithms, which may be imperfect in distinguishing multiple close fixations from one long fixation.

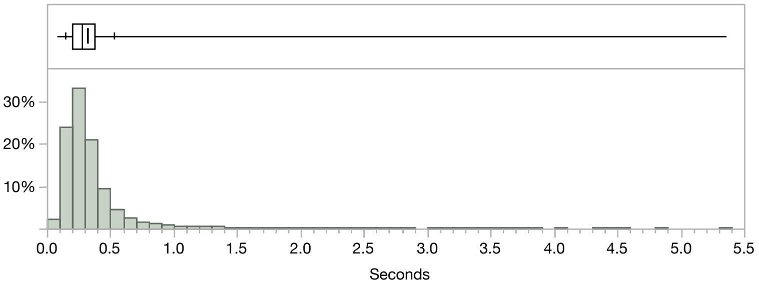

Fig. S4: Fixation durations (in seconds). Median: 0.271; mean: 0.322; inter-quartile range: 0.198-0.373. (n=53093 fixations)

A minority of participants (28 of 117) marked features in these trials. These tended to be associated with longer fixations. The median fixation duration immediately prior to creating or deleting a feature was 0.7 seconds. When fixation duration was longer than 2 seconds, the next event was more likely to be a keyboard interaction than a fixation, even though 98% of events are fixations.

Fig. S5 shows distributions of saccade durations (times between fixations). The distribution is partitioned according to whether the saccades are within an image or crossing between the two images. The durations of saccades between images were notably longer than the durations of saccades within images — as would be expected from the greater distances. Blinks result in a second mode visible in each distribution centered at 0.3 seconds. Although only 14% of saccades were center crossings, 31% of blinks occurred during center-crossings. Saccade times differ when comparing saccades within an image (mean 0.057 sec, median 0.018 sec, n=45419) with "crossing saccades" between left and right images (mean 0.139 sec, median 0.076 sec, n=6689). Median saccade times were not notably different for Analysis and Comparison phases, left and right images, and task types.

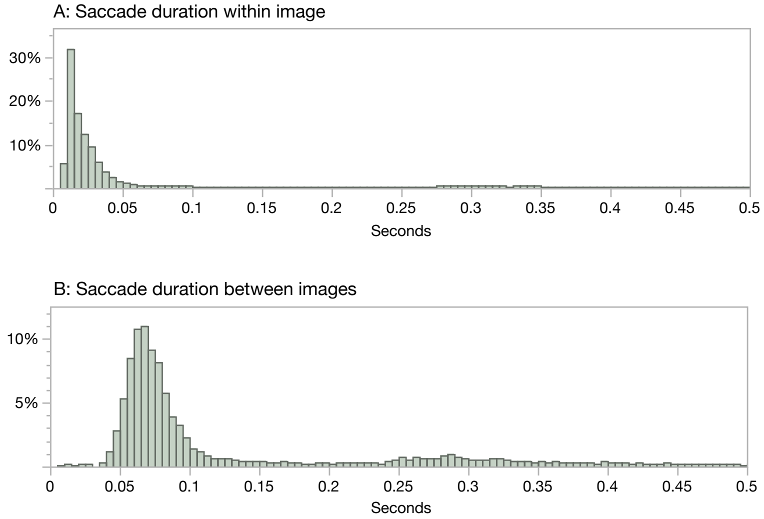

Fig. S5: Distributions of saccade durations: A) within image; B) between images. Does not show the 0.5% of saccades over 1 second. (A: n=45419; B: n=6689. Omits 310 saccades after invalid fixations.)

Appendix SI-4.2 Fixation extraction details

Fig. S6 shows an example of how eye speed varied over a one-second duration. Each 1KHz sample is labeled as belonging to a fixation (red) or saccade (blue) based on the clustering algorithm. Typical of this data, we see corrective minor saccades immediately following the fastest portion of each saccade; the algorithm tended to label these as part of the fixation, as shown here.

Observations:

- Labeling of saccades and fixations is somewhat arbitrary at this level of time resolution.
- The corrective motion is evidently based on visual information processed during what we have labeled as the saccade – prior to the eye fully settling on the final fixation location.
- The corrective saccades typically span a total distance of no more than a few ridge widths.
- Clustering this corrective movement as part of the fixation contributes some imprecision to the estimation of the fixation centroid (usually less than one ridge width).
- The labeling algorithm punctuates fixations well: most saccades are correctly identified.
- Examiners often blinked during the first left-right saccade after the right image was presented resulting in a brief gap in sampling data (not shown).

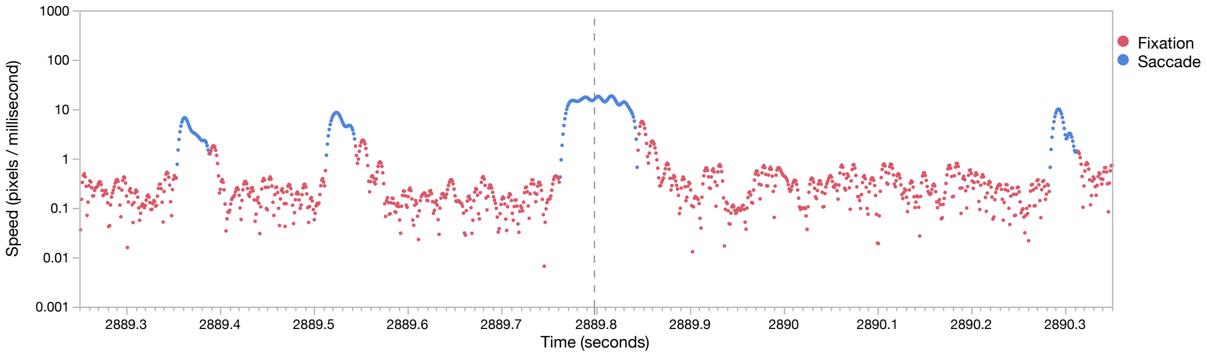

Fig. S6: Example of eye movement speed over 1.1 second interval showing labeling of saccades and fixations. The long central saccade was a transition from the left image to the right image (indicated by vertical dashed line). The images were being viewed at 1:1 zoom level (one screen pixel to one image pixel).

Appendix SI-5 Test yield and omitted data

This section provides information supporting Section 2.7 in the main document.

Out of 677 total trials, two were omitted: one in which the eye tracker lost calibration within the first second of the trial, and one with a single recorded Comparison phase fixation, resulting in 675 valid trials (219 latent, 228 plain, 228 cropped).

We omitted approximately 1% of fixations as invalid, for these reasons:

- Outside the image
- In the on-screen area containing text (The current zoom levels and markup mode were displayed in the upper-left portion of the monitor)
- In blank areas of the images (i.e. outside of the fingerprints)
- Fixations in one trial after an extremely long data gap (3 minutes), apparently due to fixations captured after completion of the task

Appendix SI-5.1 Number of fixations

Fig. S7 shows distributions of the number of valid fixations per trial, by task type, image, and phase.

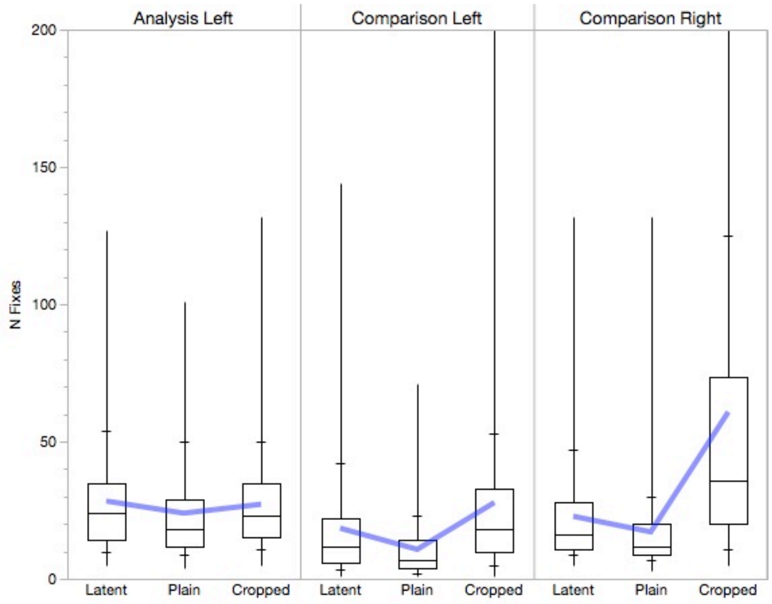

Fig. S7: Distributions of number of fixations per trial, by image and phase. Means are indicated with connecting blue lines. (n=53093 fixations, all trials included) Crossbars indicate deciles.

Appendix SI-6 Spatial analyses

This section provides information supporting Section 3.1 in the main document.

Appendix SI-6.1 Fingerprint images with fixations

Fig. S8 through Fig. S13 depict the fixations made by all examiners on image sets FT1 through FT6. The left cropped images are not shown after Fig. S8 because they are all essentially the same as in Fig. S8: fixations fill the cropped area, with some just outside the cropped area.

The color coding indicates the proportion of examiners who had any fixations in each area of the print, where areas were defined by a grid that partitioned the image into 75x75 pixel cells — one quarter the size of the target area. The grid is aligned to the corners of the target area, such that the 150x150 pixel target always contains four grid cells.

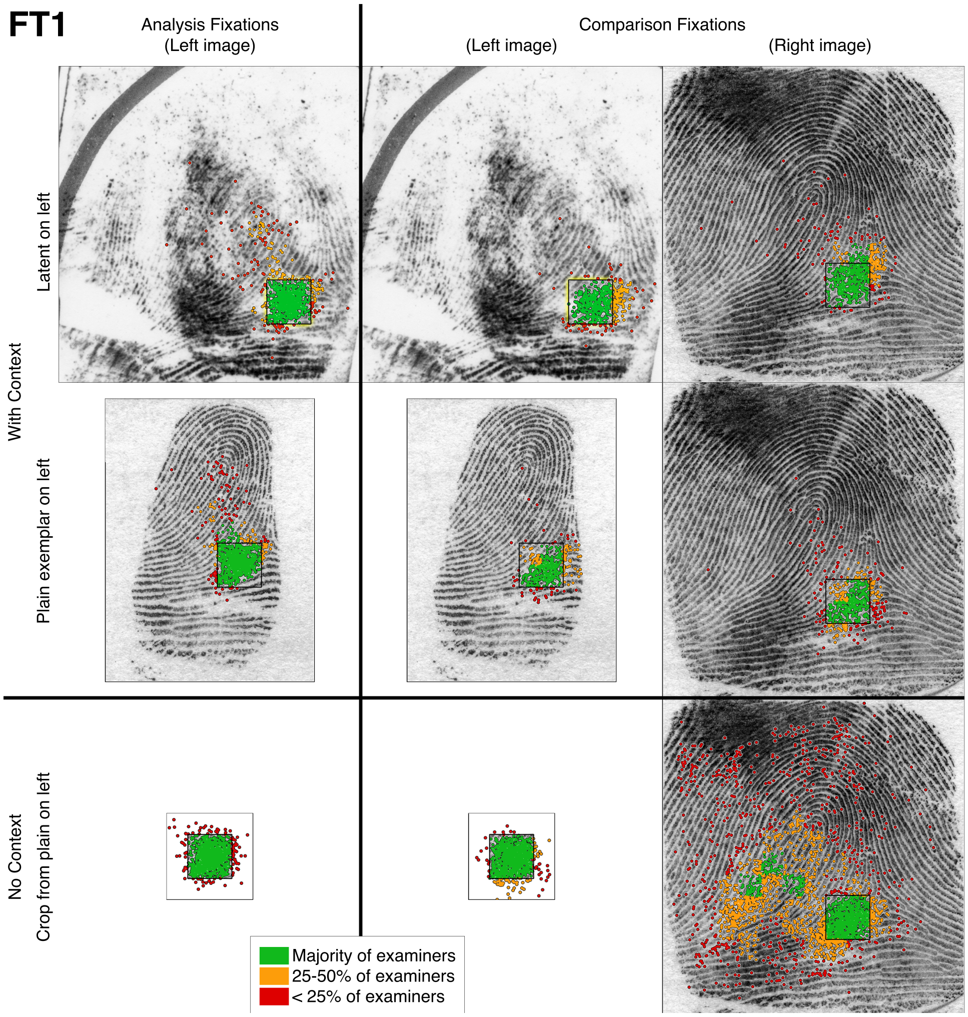

Fig. S8: Image set FT1 with fixations from all examiners. (Same as Figure 4 from the main document, but with Analysis-phase fixations)

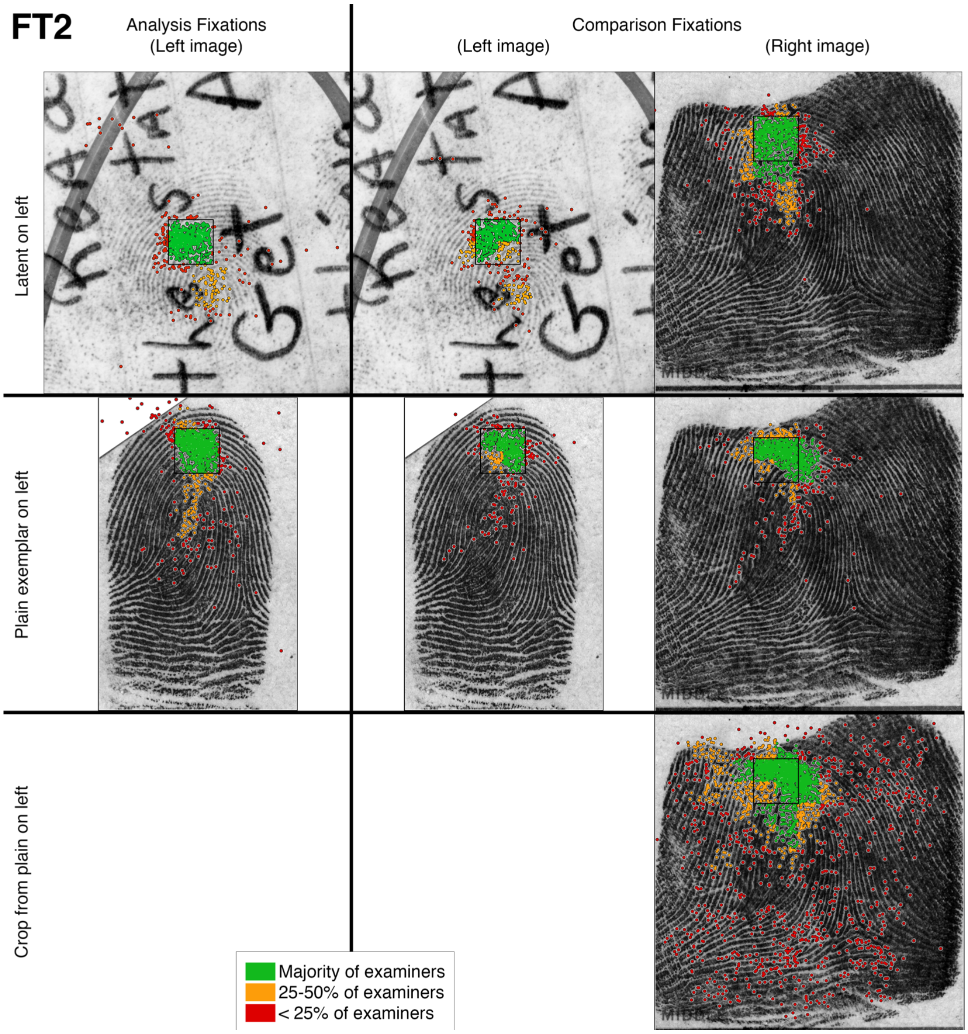

Fig. S9: Image set FT2 with fixations from all examiners.

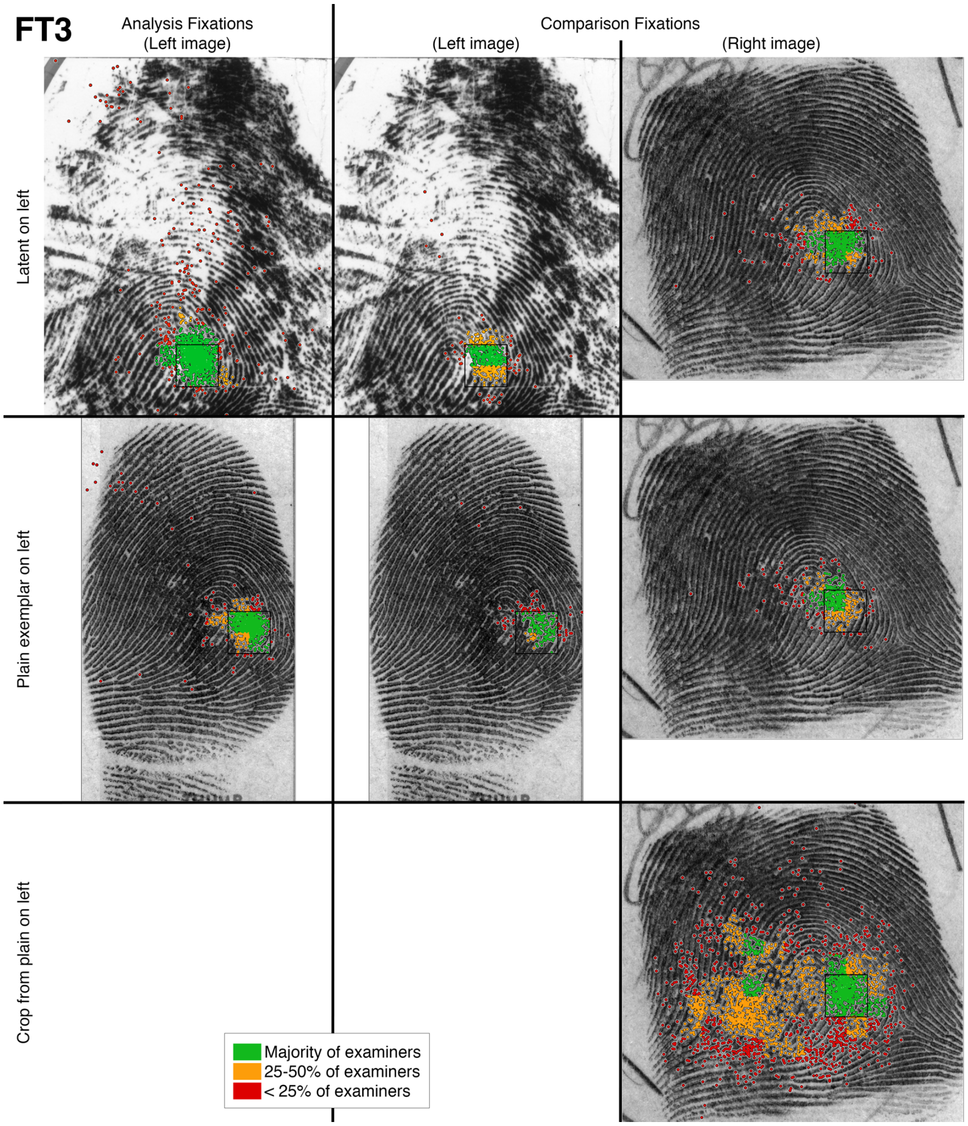

Fig. S10: Image set FT3 with fixations from all examiners.

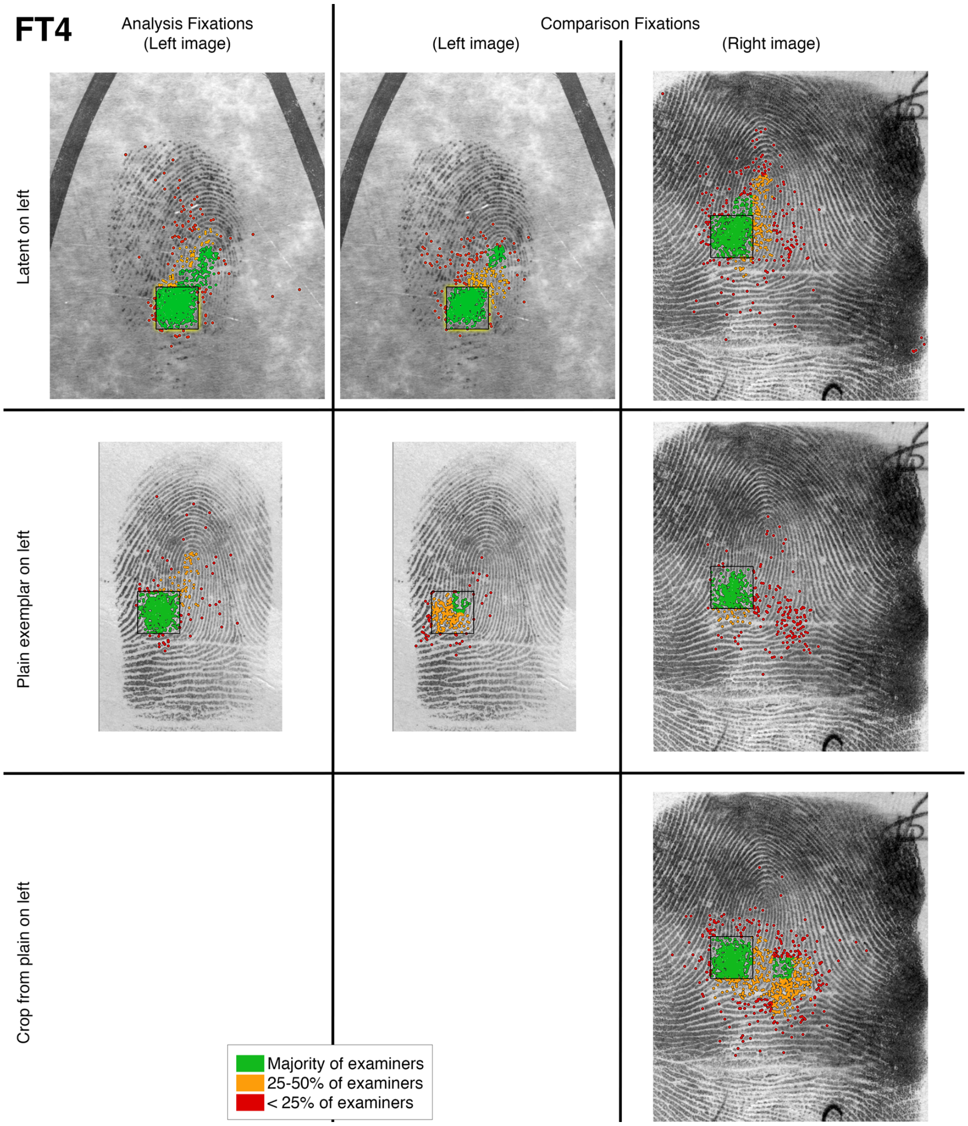

Fig. S11: Image set FT4 with fixations from all examiners.

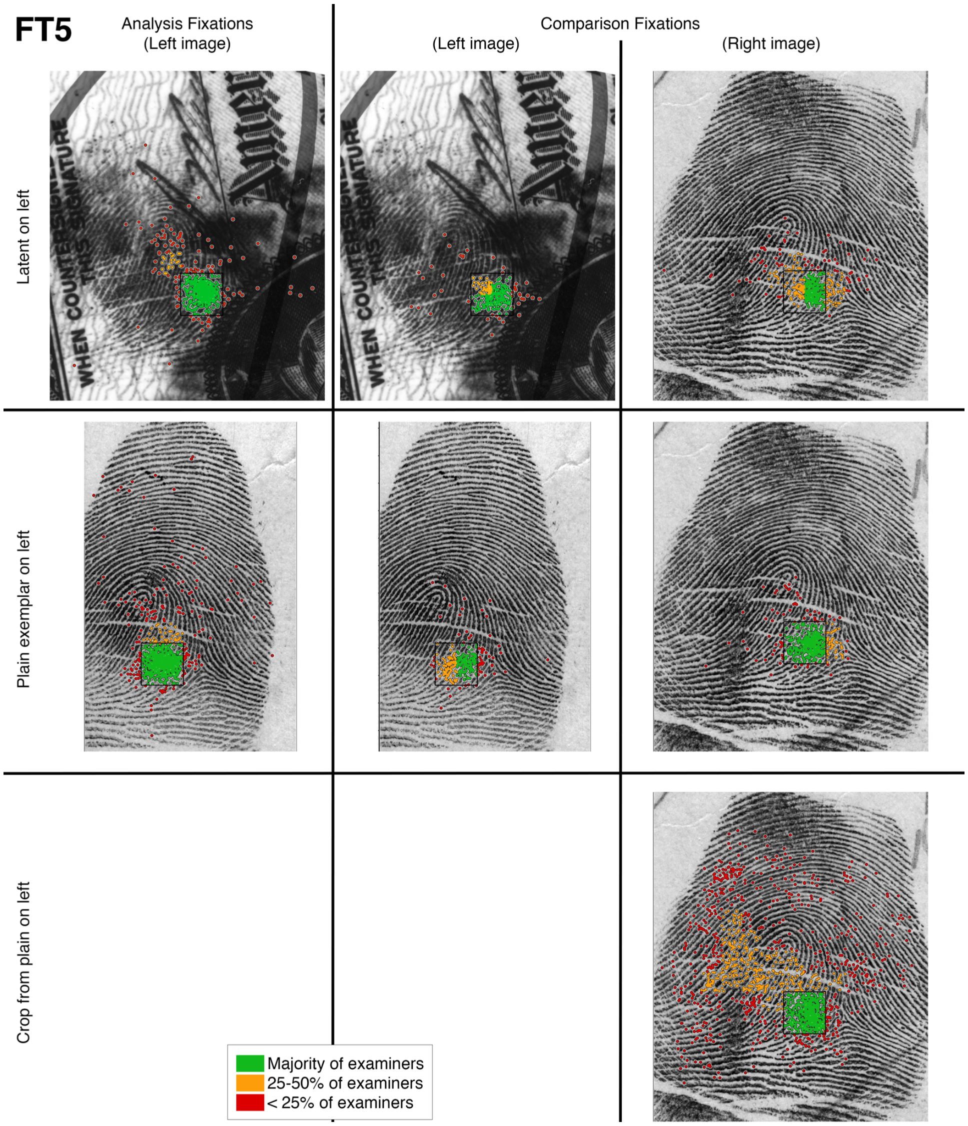

Fig. S12: Image set FT5 with fixations from all examiners.

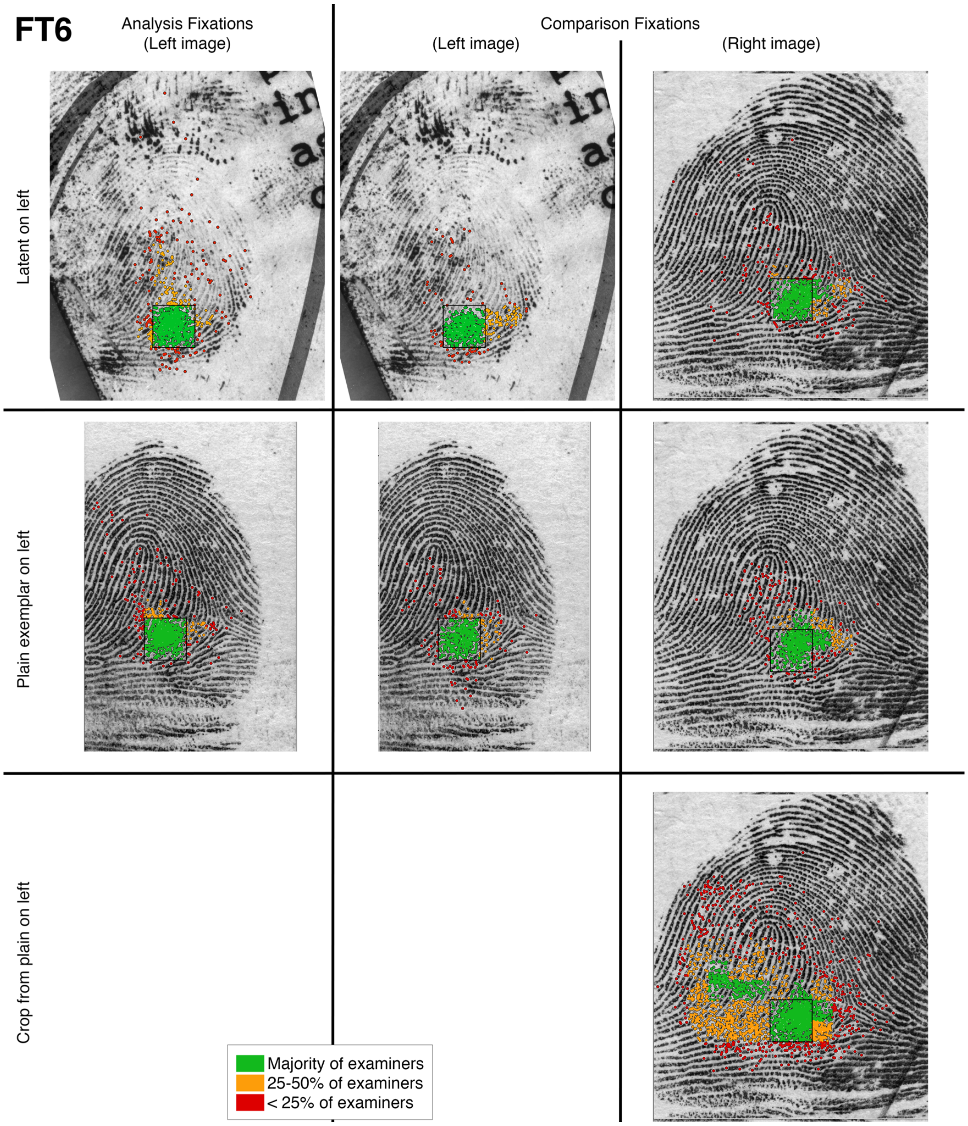

Fig. S13: Image set FT6 with fixations from all examiners.

Appendix SI-6.2 Distance from target

This section provides information supporting Section 3.1 in the main document.

Fig. S14 shows the distributions of fixations by distance from the target. The Comparison-phase charts (center and right) summarize the data presented in Figure 5.

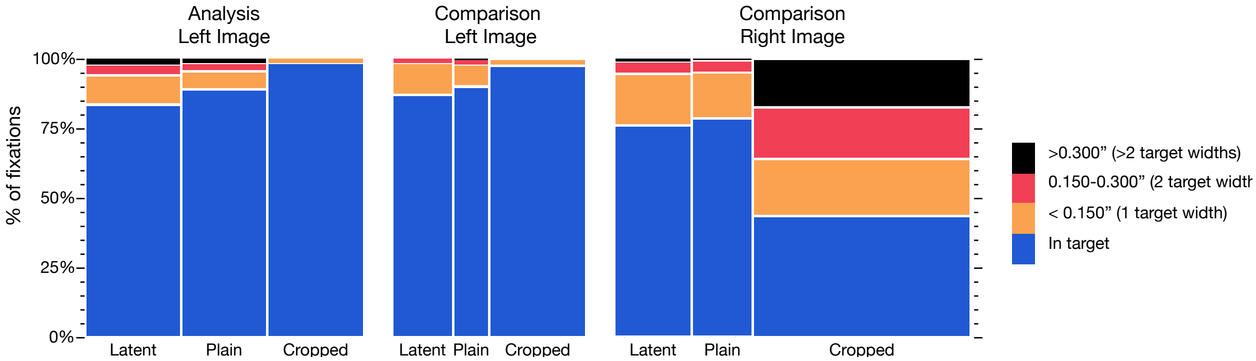

Fig. S14: Fixations by distance from target. (n=17850 fixations (Analysis left); 12496 (Comparison left); 22747 (Comparison right); all trials included)

Appendix SI-6.3 Proportion of image areas visited

This section provides information supporting Section 3.1 in the main document.

The overall dimensions of the images varied (except for the left images in Cropped tasks), as shown in Table S1. The area of ridge detail (fingerprint area) within each image was determined by manual outlining by the authors; for some latent fingerprints without clear impression boundaries, this is necessarily an approximation.

		Overall image dimensions (pixels @ 39.4ppmm)			Mean area of ridge detail as % of image	Mean image area		Mean area of ridge detail	
		Width	Height	Mean		Sq in	Sq mm	Sq in	Sq mm
Latent	Left	1120-1621	1108-1537	1329x1342	34%	1.78	1151	0.61	393
Plain exemplar	Left	648-780	985-1616	712x1232	64%	0.89	572	0.56	364
Cropped	Left	150†	150	150x150	100%	0.02	15	0.02	15
Rolled exemplar	Right	1001-1245	988-1345	1072x1185	84%	1.27	820	1.05	680

Table S1: Image dimensions (ranges) and mean areas of the images (rectangles) and the areas of fingerprint ridge detail, by image type.

Fig. S15 describes the number of 75x75 pixel cells “visited” (fixated within) by each examiner (as described in Appendix SI-6.1); above each distribution is a red marker indicating the total number of cells visited by any examiner (on that task, phase, and image). Across all trials, the median area (number of cells) visited by examiners in the right image on cropped tasks was more than triple that of plain tasks, and nearly triple that of latent tasks. On most latent and plain trials, examiners concentrated in a small area in the right image. On the right image, examiners visited 10 or more cells on 10% of plain trials, 21% of latent trials, and 63% of cropped trials. For right images in cropped tasks, a few examiners are responsible for much of the visited area: in the most extreme case (FT2), one examiner visited 92% of the area visited by any examiners. For most images, the mean (and median) area was only about 15-20% of the area visited by any examiners.

† *In the cropped images, the area of fingerprint ridge detail was always 150x150 pixels (at 39.4ppmm), but displayed within a 300x300 white background. Fixations within the white area were not omitted.*

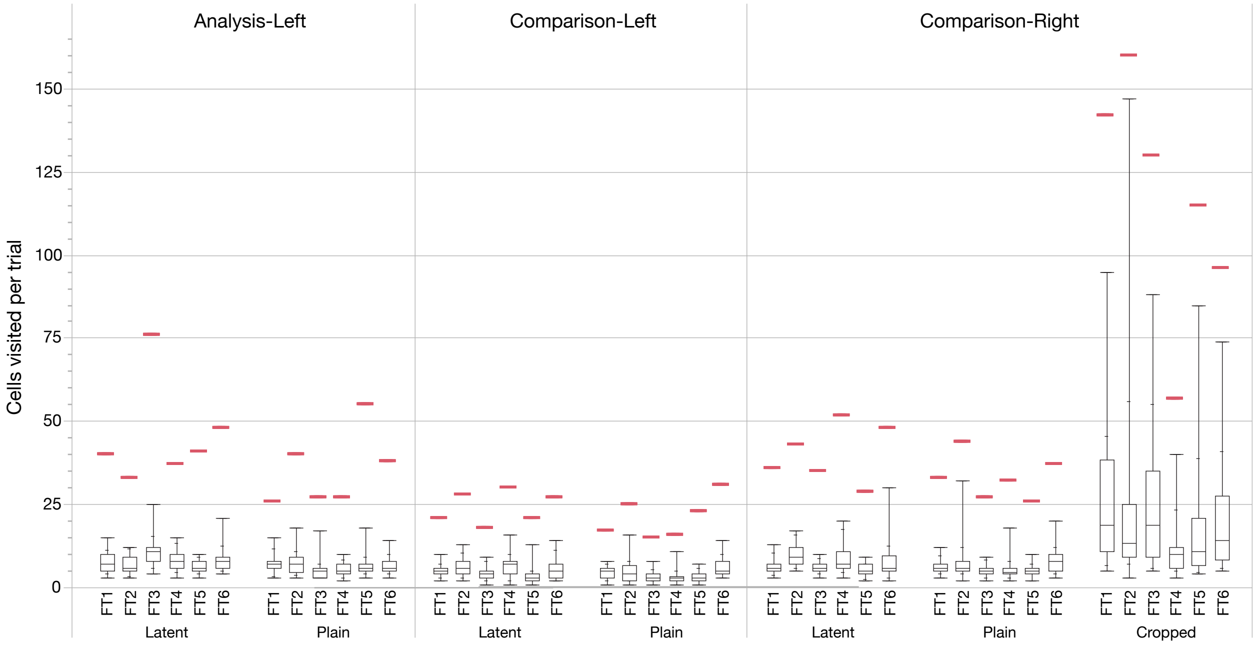

Fig. S15: Area of each image visited per trial. Area (y axis) is measured as the number of 75x75 pixel cells visited. Boxplots summarize data across all trials; red lines show total area visited by any examiners. N varies from 29 to 39 examiners (mean=35).

Examiners generally focused their attention in a small area within each image. Fig. S16 describes the distribution of fixations throughout those areas of the images that contained ridge detail. Except for cropped right images, most cells had no fixations from any examiner; only a small percentage of cells had fixations from a majority of examiners.

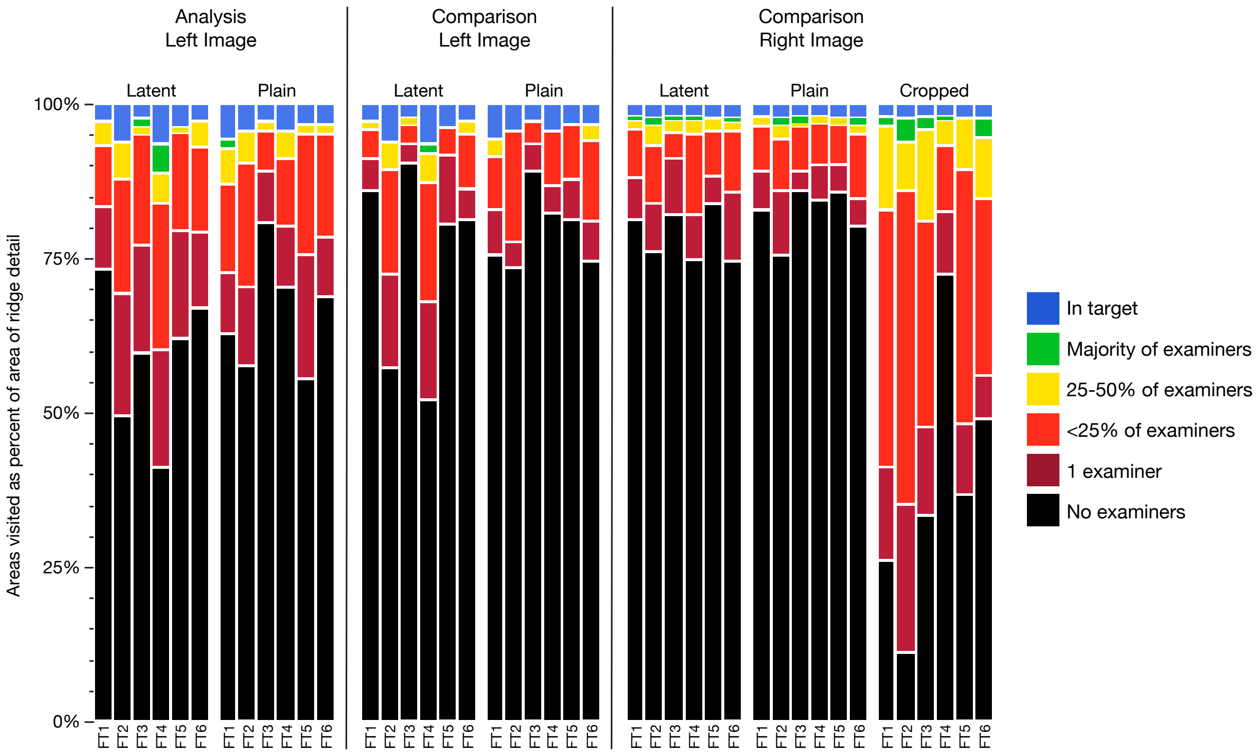

Fig. S16: Proportions of total area of ridge detail visited by examiners in each image. The areas visited by examiners are based on the number of examiners who had any fixations in 75x75 pixel grid cells. The area of ridge detail (i.e. fingerprint area) in each image was manually determined by the authors. Cropped left images are omitted because the area of ridge detail and target were identical.

Appendix SI-6.4 Distance to core

Fig. S17 shows how close each examiner looked to the core in each trial (i.e. the closest fixation to the core in each trial). If we consider that a fixation within about 4 ridges of the core (< 2.24mm) is “close” and more than about 8 ridges (>4.47mm) is “far,” then examiners generally looked close to the core in Analysis, and in Comparison on the right image for cropped tasks; however, in most trials the closest fixations were still far from the core in Comparison in the left image for latent and plain tasks.

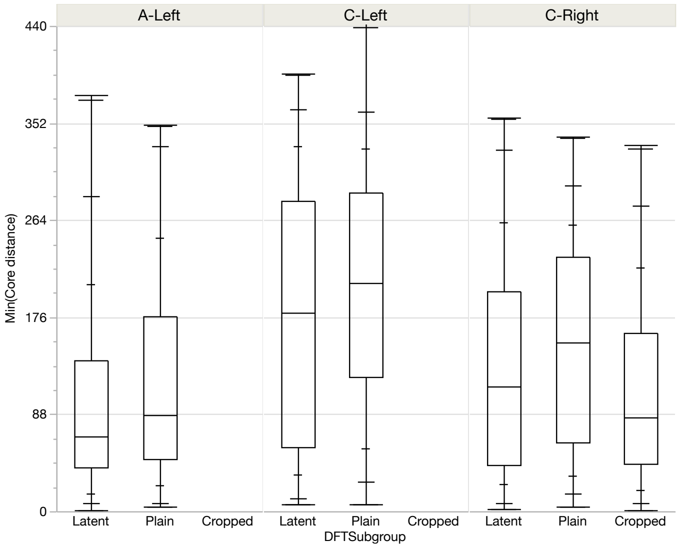

Fig. S17: Minimum distance to core, by trial type, phase, and image (left or right). (n=675). "Core" is defined as uppermost point on innermost recurving ridge. Distances in pixels (at 39.4ppmm): 88 pixels (2.24mm) is on average about 4 ridges.

Appendix SI-7 Time to complete

This section provides information supporting Section 3.2 in the main document.

Appendix SI-7.1 Overall phase and trial duration

Fig. S18 shows median Analysis and Comparison durations for each image set and task type. Median Analysis durations for each image set were consistently lower on the plain tasks than on the cropped tasks, but for latent tasks varied greatly by image set. Although the median Comparison duration for all cropped trials was nearly twice as long as for latent trials, and three times as long as for plain trials, these ratios varied notably by image pair. For example, in FT4, the median Comparison duration of latent tasks was triple that of plain tasks, whereas in FT5 the median Comparison duration of latent tasks was less than that of plain tasks. (We define the start of Comparison to be the start of the first fixation in the right image after the right image was shown.)

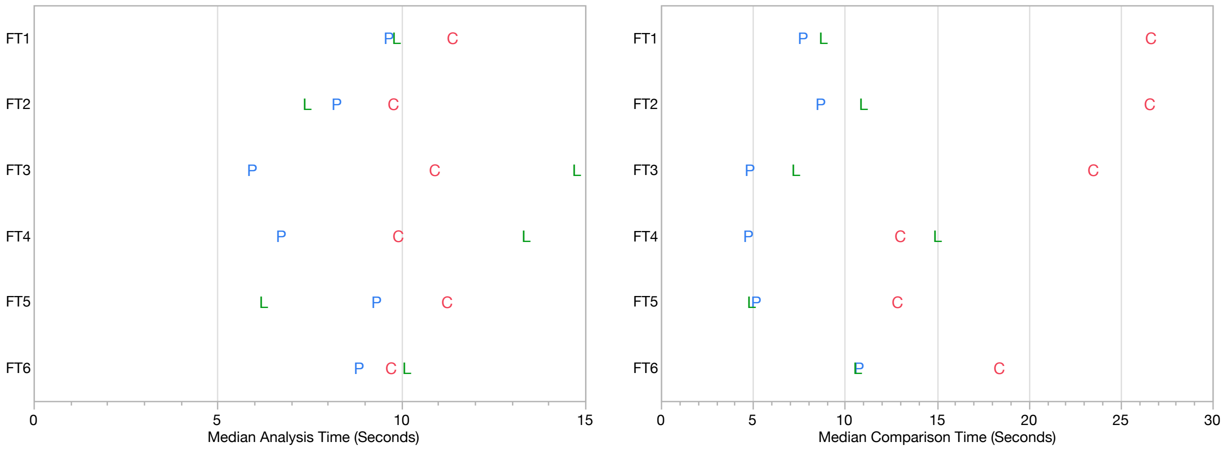

Fig. S18: Median durations of Analysis and Comparison phases (seconds), by task type and image set. Overall median Analysis time: Latent 10.4 seconds; Plain 8.2; Cropped 10.7. Overall median Comparison time: Latent 9.5 seconds; Plain 6.1; Cropped 18.2.

Appendix SI-7.2 Effect of trial number on Analysis and Comparison durations

We observed a learning effect: Analysis and Comparison durations tended to decrease over successive trials.

Analysis durations were longest for the first find-the-target trials and tended to decrease over successive trials, as shown in Fig. S19. Most examiners completed six find-the-target trials (89% completed at least 5 trials). Overall, the median duration spent on Analysis dropped from 18 seconds on the first trial to 5 seconds by the 6th trial.

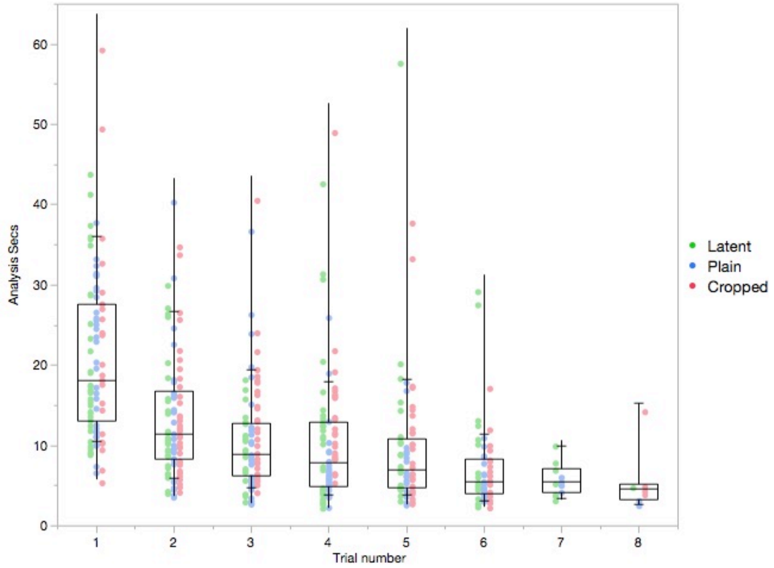

Fig. S19: Analysis time as a function of trial number. There was no notable effect by task type (color coding). (N varies by trial number and task type: for 1st trial, n=41 on latent and plain, 35 on cropped, dropping to 30,35,26 on 6th trial.) Crossbars indicate deciles.

Comparison times did not show as strong a learning effect as did Analysis times. Because behavior during Comparison is sensitive to the type of task, and most examiners had only two trials of each type, we observe the learning effect over two trials, not eight. Table S2 shows the drop in median Comparison times from the first to the second trial of each task type.

	Median Comparison duration (seconds)		(%)
	1st trial of this type	2nd trial of this type	1st trial was longer
Latent	10.1	8.9	59%
Plain	6.2	5.4	57%
Cropped	24.2	13.7	69%

Table S2: Effect of trial number on median Comparison times, and the proportion of examiners for which the first trial of each task type was longer than the second trial of the same type. N=614 trials (omits 36 trials for which the examiner only completed one trial of that type, and 25 trials that were the third of a type for that examiner).

Appendix SI-7.3 Relations between Analysis and Comparison times

Trials with longer Analysis times tend to have longer Comparison times, as summarized in Fig. S20. The correlation is strongest for cropped trials, in part because on the other trials Comparison time was often very short regardless of Analysis time.

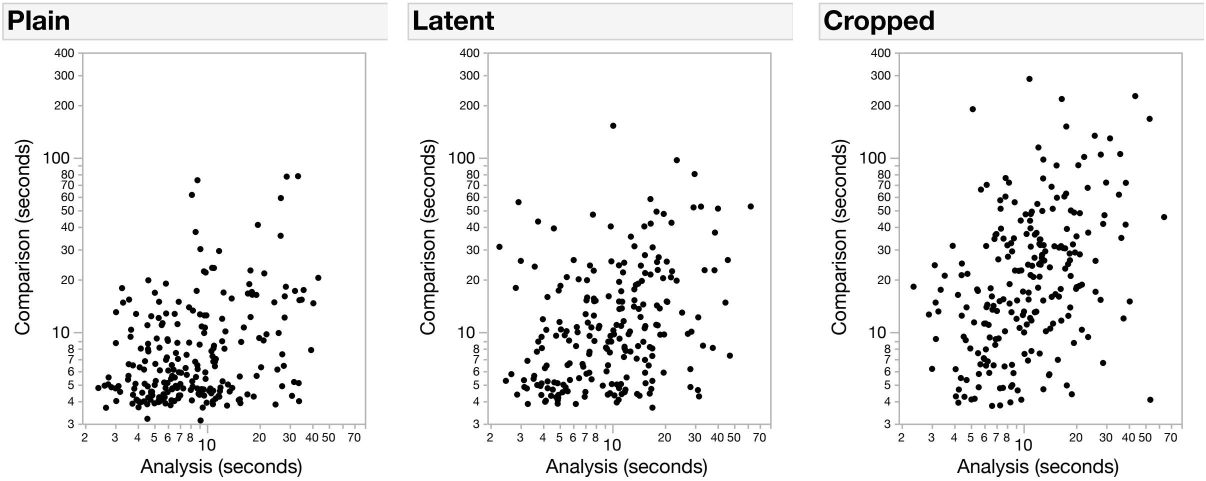

Fig. S20: Analysis vs. Comparison time by left image type. R^2 for Log(Analysis time) vs. Log(Comparison time): 0.1142 (latent); 0.1158 (plain); 0.1670 (cropped). (n = 219, 228, 228 trials)

Appendix SI-7.4 Factors accounting for variability in Analysis and Comparison times

As shown in Table S3, three factors accounted for much of the variability in log(total time) spent on trials: examiner, trial number, and type of task. The trial number was an integer describing the ordinal position of this trial among all FT trials that each examiner completed. Much of the variability in Analysis time can be accounted for by examiner and trial number; for Comparison time much of the variability can be accounted for by examiner and task type (shown in bold).

**Independent variables**	**Adj. R^2 Log(Analysis)**	**Adj. R^2 Log(Comparison)**
Examiner	0.309	0.332
Examiner + Log(Trial_Num)	**0.627**	0.353
Examiner + Task_type	0.331	**0.555**
Examiner + Log(Trial_Num) + Task_type	0.646	0.573
Log(Trial_Num)	0.317	0.026
Task_type	0.017	0.201
Log(Analysis_secs)	n/a	0.142
Log(Analysis_secs) + Task_type	n/a	0.304
Examiner + Log(Analysis_secs) + Task_type	n/a	0.588

Table S3: Comparison of logistic regression models predicting Analysis and Comparison time spent on each trial. Examiner was modeled as a random effect; the other factors were modeled as fixed effects.

Appendix SI-8 Rapid localization

This section provides information supporting Section 3.3 in the main document.

Examiners frequently fixated in the target area of the right image within the first few fixations after the right image was presented. Fig. S21 shows that examiners looked in the target area with the first right fixation in 23% of latent trials and 36% of plain trials, but only 7% of cropped trials; examiners looked in the target area by the fourth right fixation in 89% of latent trials, 95% of plain trials, and 50% of cropped trials.

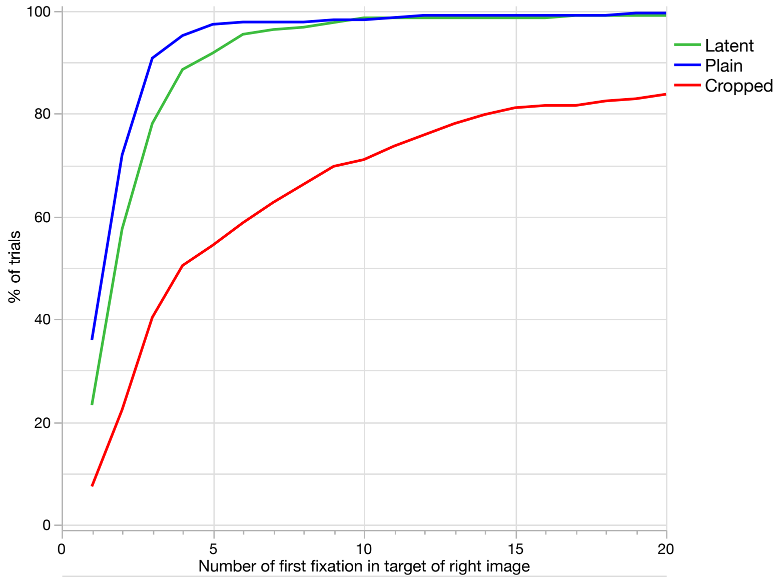

Fig. S21: Cumulative plot: proportion of trials by the number of the first right fixation in target (counting from when the right image was presented). (n=675 trials)

Fig. S22 uses raw (1KHz) data to describe which images, left or right, examiners were focusing on during the first three seconds after the right image was presented. This result was not sensitive to the task type. The first eye crossings to the right image began abruptly approximately 150 milliseconds after the right image was presented. Approximately one half of examiners first crossed to the right image within 250 milliseconds. At approximately 500 milliseconds, a few examiners had crossed back to the left image. After two seconds, the majority of examiners had looked to the right and back to the left again (green and yellow); 16% of examiners had looked to the right, to the left, and to the right again (yellow).

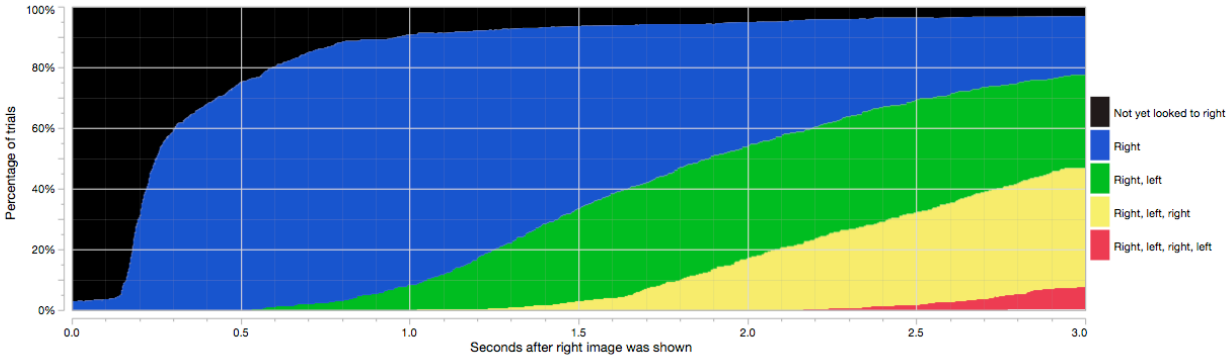

Fig. S22: Which images examiners looked at within 3 seconds after the right image was presented. Black: examiner had not yet looked to the right image at the specified time; Blue: examiner had looked at the right image; Green: examiner looked from left to right to left image; etc. for Yellow and Red. In 1% of trials (too small to be visible) the examiner looked right-left-right-left-right within the first three seconds. (n=675 trials).

Fig. S23 provides supporting information for Section 3.3 in the main document, showing the distributions of localization times by image pair and task type; these distributions are summarized as medians in Figure 7.

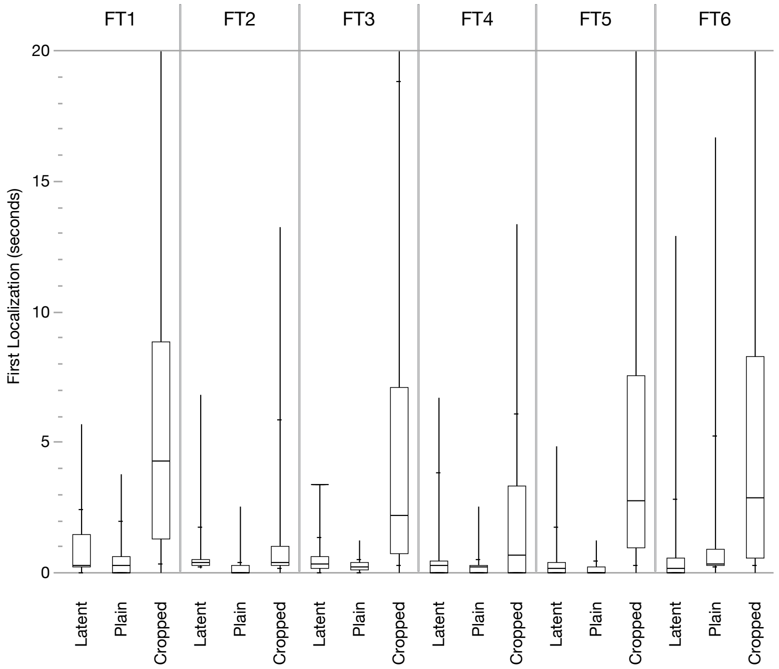

Fig. S23: Distributions of localization times by image pair and task type. Full distributions of data summarized as medians in Figure 7. Data truncated at 20 seconds. (n=626 trials; 4 trials had no localizations).

Appendix SI-9 Errors finding the target

This section provides information supporting Section 3.4 in the main document.

Fig. S24 through Fig. S26 show the right-image fixations made on six trials where the examiner apparently failed to find the target, as discussed in Section 3.4. In the four apparent errors shown in Fig. S24 and Fig. S25, most fixations were far from the target, and there was at most one fixation in the target. In the two apparent errors shown in Fig. S26, although the examiners did look in the correct target area, most fixations were far from the target, and they did not look in the target near the end of the trial, strongly suggesting that they had incorrectly decided the target was in another area.

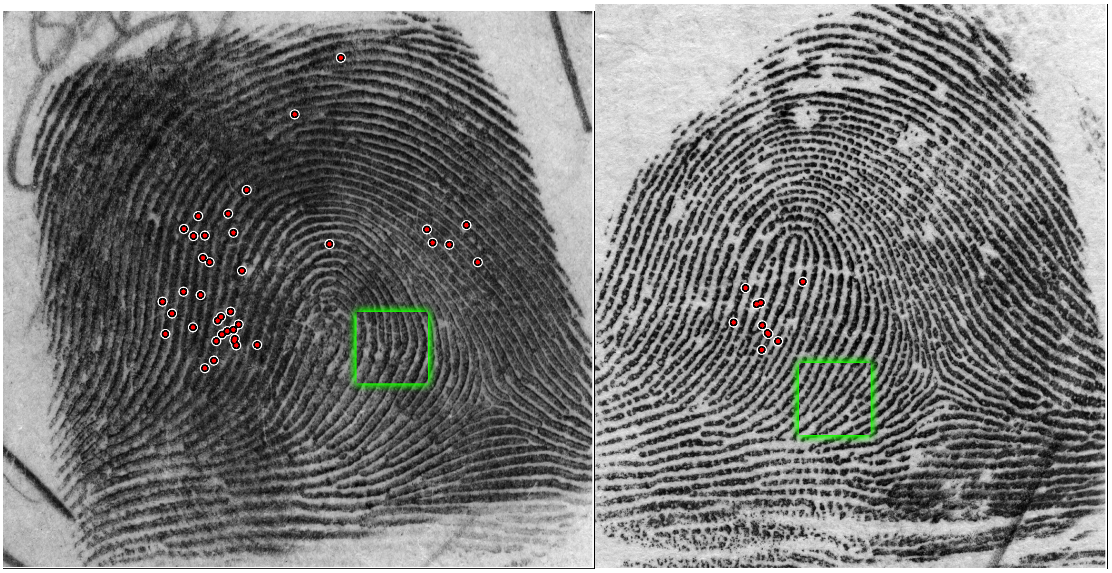

Fig. S24: Errors on cropped tasks (left: FT3, right: FT6) — no fixations in target. These two trials were from the same examiner.

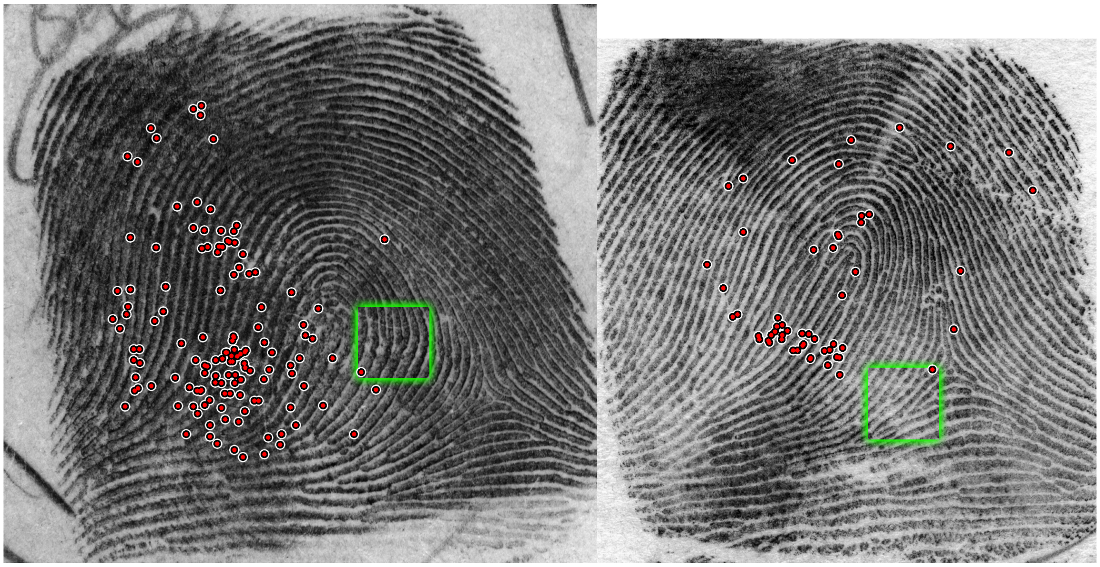

Fig. S25: Errors on cropped tasks (left: FT3, right: FT1) — one fixation in target, and no fixations in target in last 10 seconds. The trial on the left had an additional fixation in the 30-pixel margin around the target.

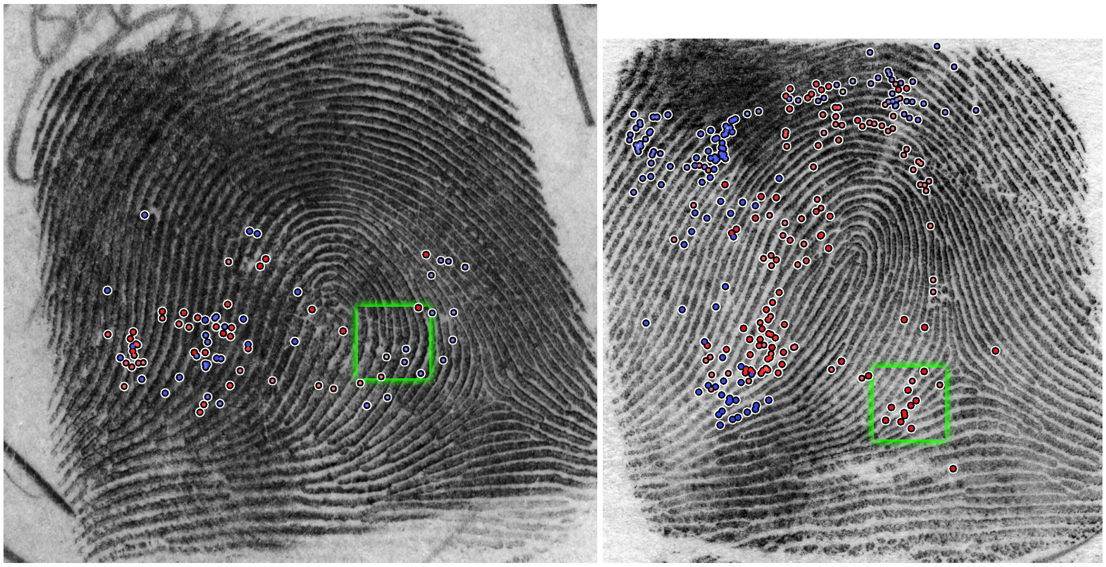

Fig. S26: Errors on cropped tasks (left: FT3, right: FT1) — no fixations in target in last 10 seconds. Fixation colors are a gradient by Comparison time, from red at start of Comparison to blue at the end of Comparison. Details for left trial: 9 fixations in target (including 30-pixel margin), 74 fixations outside; last fixation in target ended 21.9 seconds in Comparison phase of 36.2 seconds. Details for right trial: 14 fixations in target (including 30-pixel margin), 252 fixations outside; last fixation in target ended 37.4 seconds in Comparison phase of 100.8 seconds.

Appendix SI-10 Timelines

This section provides information supporting Section 3.5 in the main document.

Fig. S27 through Fig. S29 show timelines for all trials on image sets FT2-FT6 (equivalent to Figure 9, which shows FT1). Fig. S30 shows the detailed view of the last 10 seconds of Comparison for all trials on image sets FT2-FT6.

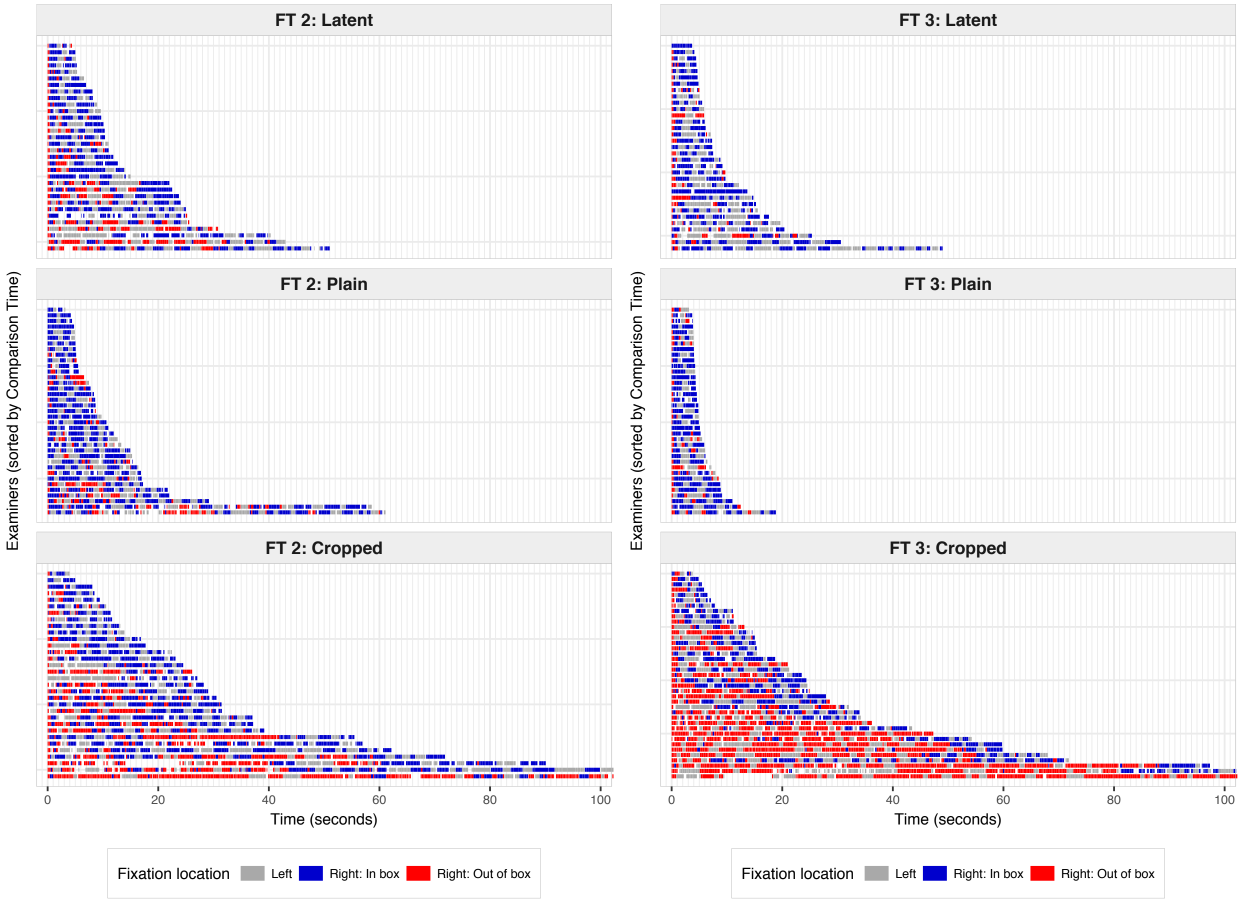

Fig. S27: Timelines for all trials on image sets FT2 and FT3. Comparison times truncated at 100 seconds.

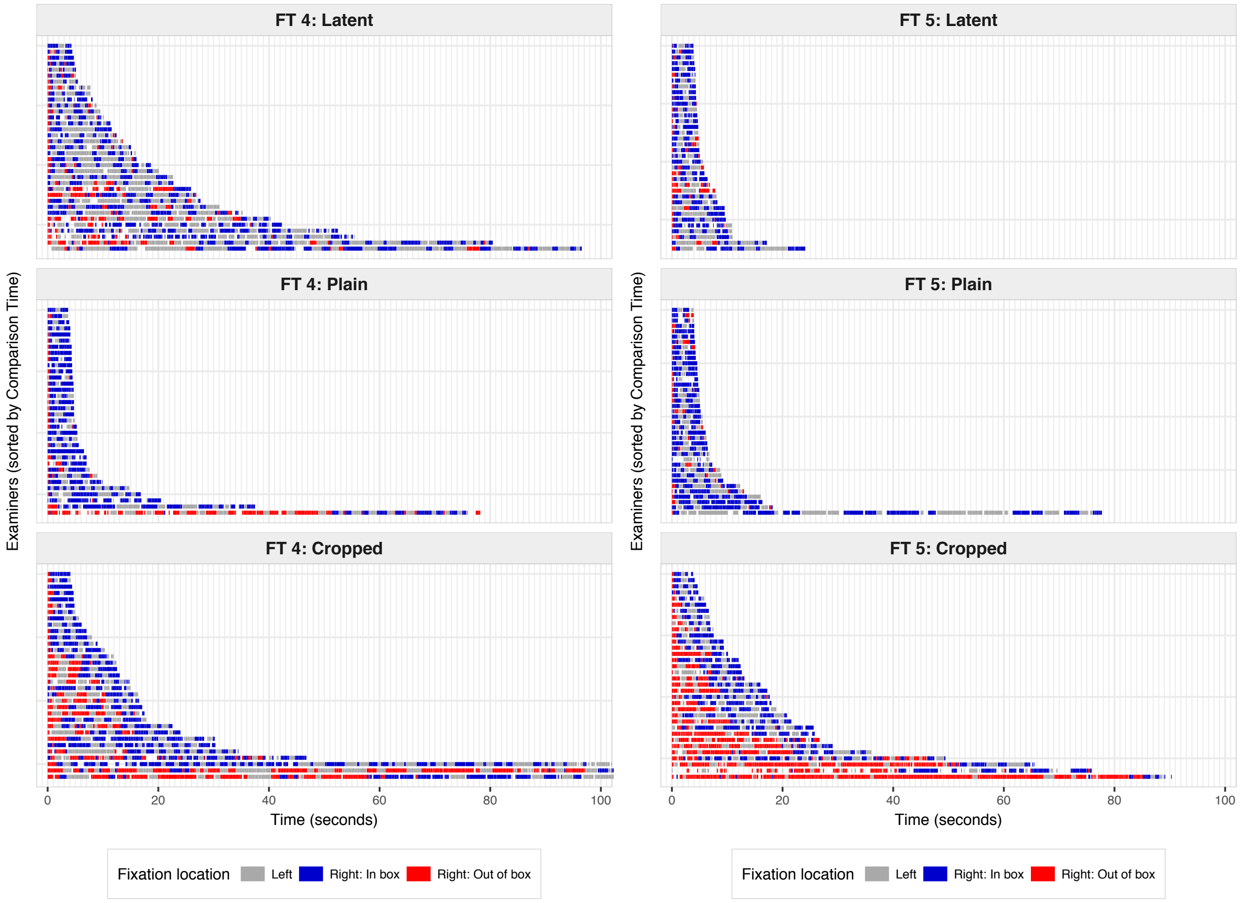

Fig. S28: Timelines for FT4 and FT5. Comparison time truncated at 100 seconds.

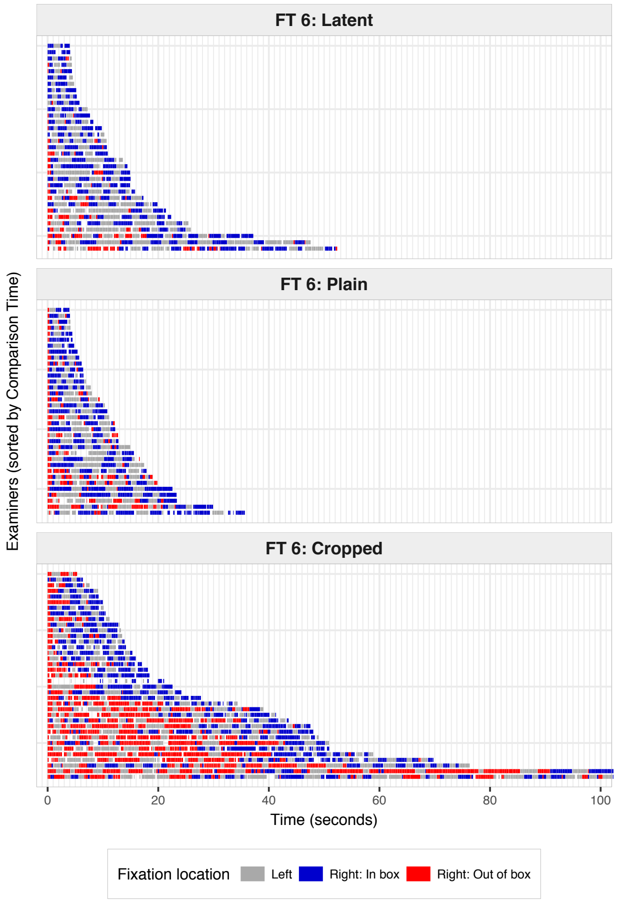

Fig. S29: Timelines for FT6. Comparison time truncated at 100 seconds.

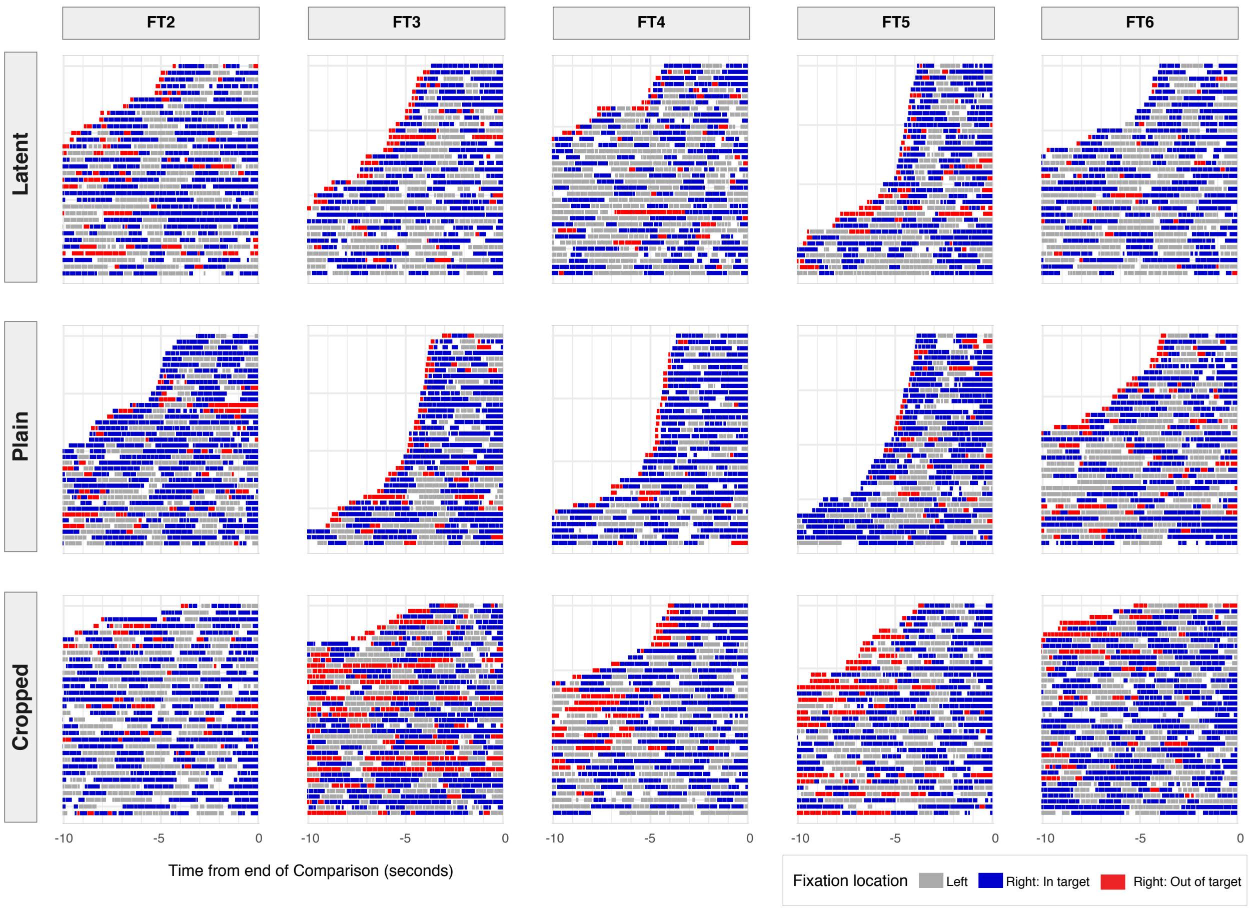

Fig. S30: Last 10 seconds of Comparison phase timelines for all trials on image sets FT2-FT6 (FT1 is in Figure 9 of main paper).

Appendix SI-11 Total time in the target

This section provides information supporting Section 3.5 in the main document.

Typically, examiners fixated in the correct target area in the right image for a total of about three to six seconds (inter-quartile range of plain or latent trials) or four to eleven seconds (inter-quartile range of cropped trials) before completing the trial, as shown in Fig. S31.

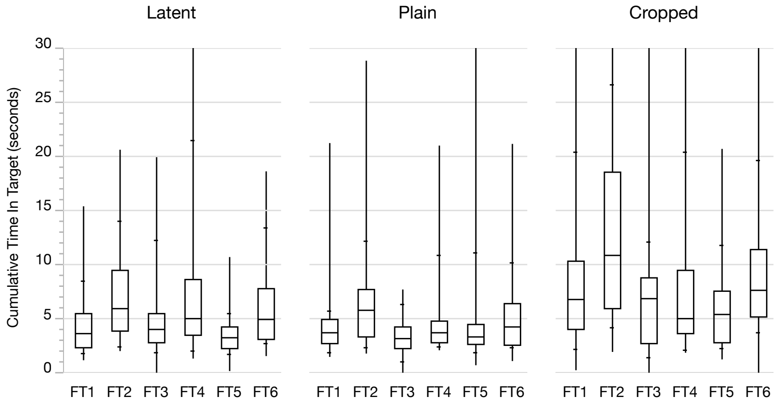

Fig. S31: Distributions of total time in right target area, calculated as the sum of fixation durations, including saccade durations between fixations in the target area. Medians: 4.3 seconds for latent tasks; 3.6 for plain; 6.5 for cropped. (n=630 trials, omitting FT7-FT8)

Appendix SI-12 Subphases

This section provides supporting information to Section 4 in the main document.

Fig. S32 illustrates how we partition timelines into subphases, using the color-coding from Figure 9, in which blue indicates in target in the right image; red, out of the target in the right image; and gray, left image.

- Subphase A is the period at the ***start*** of Comparison prior to the examiner first looking at the target, in which all fixations in the right image are outside the target. For trials in which the first right fixation is red (out of the target), subphase A is the period prior to the first continuous 0.5 second of blue (in).
- Subphase C is the period at the ***end*** of Comparison when the examiner is only looking at the target, in which all fixations in the right image are in the target in the right image. For trials in which the last right fixation is blue (in the target), subphase C is the period after the last 0.5 second of red (out), until the end of the trial; for trials that have no red, the entire Comparison phase is considered subphase C.
- Subphase B is simply the remainder: any period of time not in subphase A or C.

Subphase A is therefore the period before the examiner finds the target, subphase C is the period after the examiner stops considering any areas outside the target, and subphase B is the intermediate period between.

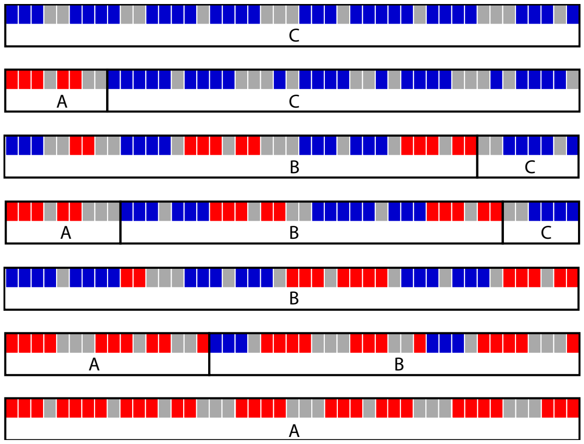

Fig. S32: Illustration of how trials are partitioned into subphases (hypothetical data in which all fixations have the same duration (1/3 second)).

Fig. S33 shows all trials, colored by subphase. Notice that subphase A is generally brief in latent and plain tasks but often lengthy in cropped tasks, subphase B is most prevalent in cropped tasks, and subphase C is present in almost all tasks.

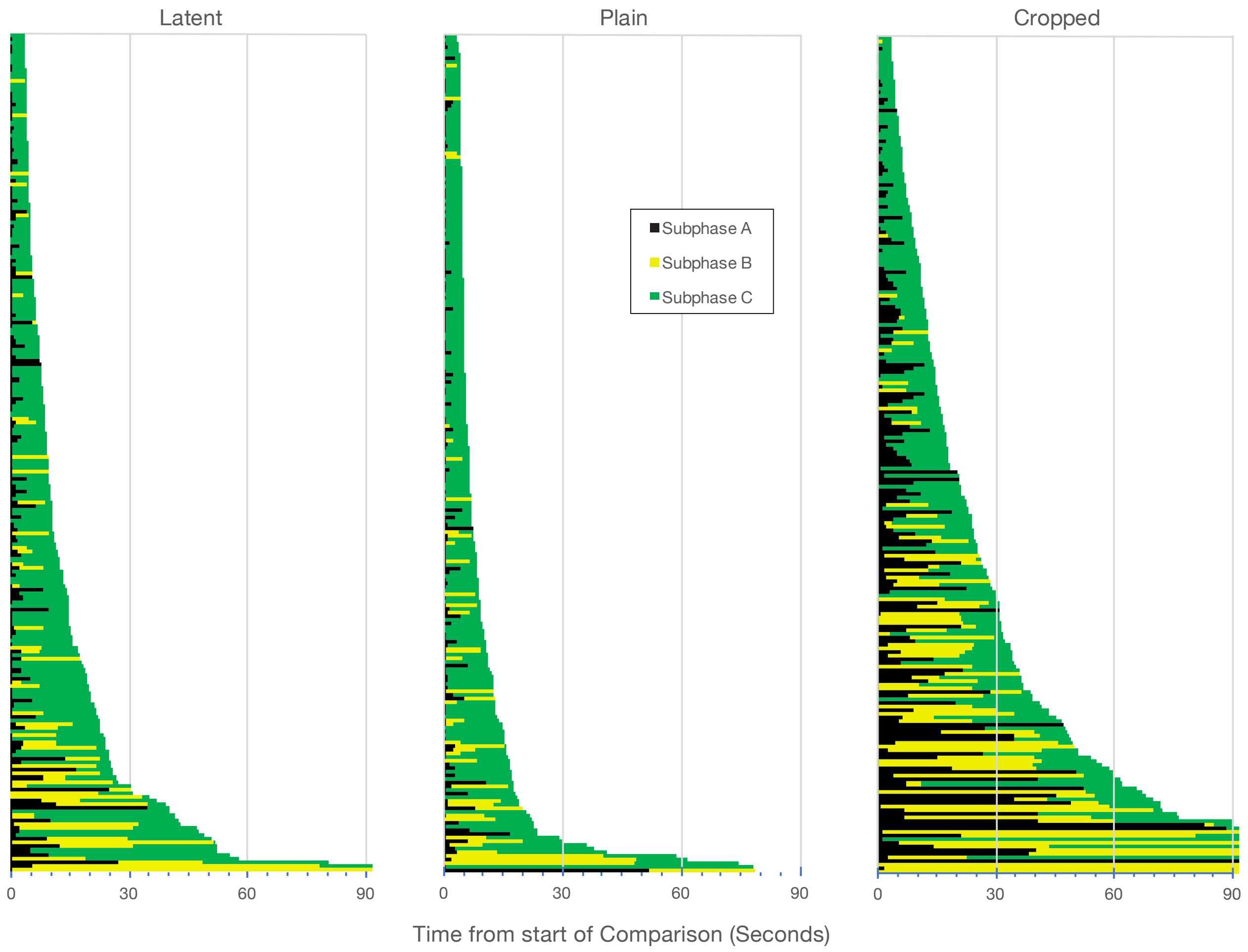

Fig. S33: Timelines for all 675 trials, color-coded by subphase. Trials truncated at 90 seconds: two latent trials (max 152 sec) and 15 cropped trials (max 281 sec).

Appendix SI-13 Overt behavior metrics

This section provides information supporting Section 4.2 in the main document.

Appendix SI-13.1 Speed3 metric

Speed3 measures the speed of eye movement within an image in image pixels per second. For each fixation, *Speed3* is measured over a series of up to ± three fixations, as the sum of inter-fixation distances, divided by the

time from the start of the first fixation to the end of the last fixation in the series. Fixations near left-right transitions will have fewer than ± three fixations in the series.

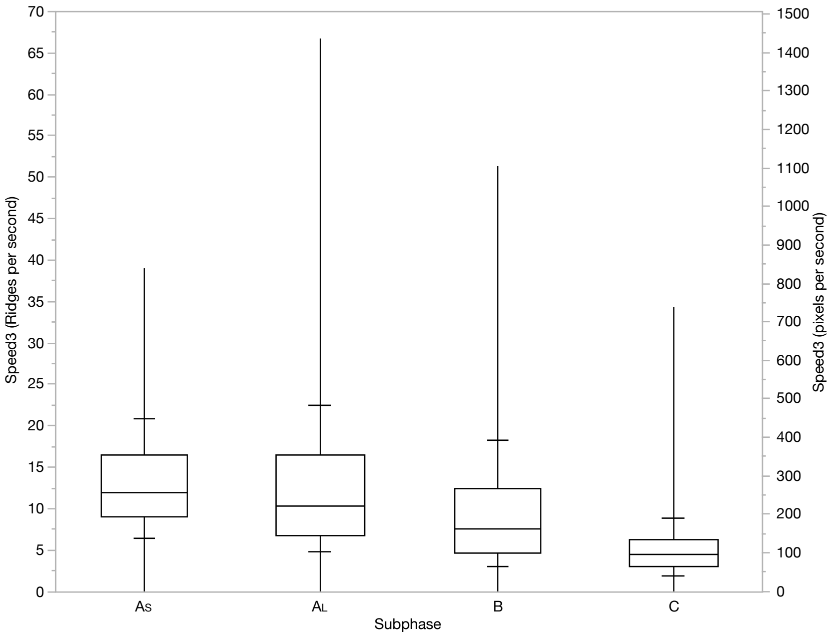

Fig. S34: Distributions of the *Speed3* metric by subphase. Medians: A_S 11.9 ridges/second; A_L 10.3; B 7.6; C 4.5. n=22747 right-image fixations. Crossbars indicate deciles.

As a different measure of the same underlying behavior, the distances between consecutive fixations are generally short in subphase C and long in subphase A (especially A_S) (medians: subphase A_S=5.5 ridges, A_L=3.2, B=2.3, C=1.7).

Appendix SI-13.2 PercentOfFixesInCell metric

PercentOfFixesInCell measures the percentage of fixations made by this examiner in a given trial that are located in each cell in a 1.9mm x 1.9mm grid. For each fixation, *PercentOfFixesInCell* is calculated for the cell in which the fixation is located. This local spatial density measure may reflect an examiner's level of interest in an area. For example, if in a trial an examiner only looks in the target area (which contains four cells), the sum of *PercentOfFixesInCell* for those four cells will total 100%. In Fig. S35, we see that in subphase C half of the fixations were in cells in which the individual examiners placed at least 21% of their fixations, whereas in subphase B the examiners were much less spatially focused, with half of their fixations concentrated in cells that received only 5% of their fixations.

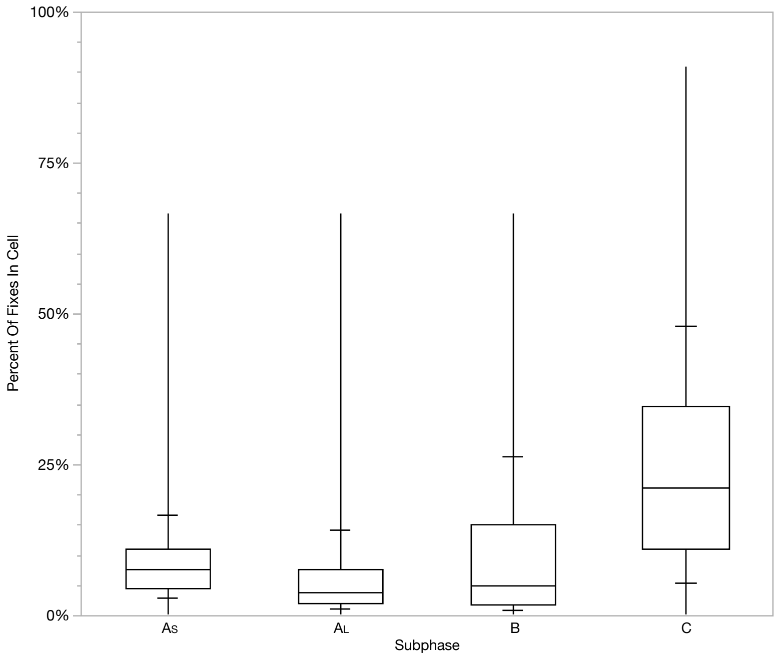

Fig. S35: Distribution of the *PercentOfFixesInCell* metric by subphase. Medians: A_S 7.7%; A_L 3.8%; B 5.0%; C 21.3%. n=22747 right-image fixations. Crossbars indicate deciles.

Appendix SI-13.3 Image visits and TimeInImage metric

We use "image visit" to refer to a consecutive series of fixations in an image (left or right), bounded by the transitions into and out of that image. *TimeInImage* is the duration of time spent in each image visit, from the start of the first to the end of the last fixation in that image. Table S4 summarizes the duration of image visits by image and task type.

	Left			Right		
	Latent	Plain	Cropped	Latent	Plain	Cropped
Mean	2.1	1.5	1.8	2.3	2.3	5.0
Min	0.1	0.1	0.1	0.1	0.1	0.1
Q1	1.0	0.8	0.8	1.1	1.2	1.8
Median	1.6	1.1	1.2	1.8	1.9	3.3
Q3	2.7	1.8	2.1	2.8	2.8	6.2
Max	10.0	12.8	13.2	13.7	10.0	29.2
n	913	636	1657	1071	804	1838

Table S4: Summary statistics for *TimeInImage* (in seconds). n=6919 Comparison-phase image visits.

Each image visit includes a series of fixations. Most image visits in the right image occur within a single subphase, but some span more than one subphase, as shown in Table S5.

Subphase(s) in image visit			Image visits	% of all image visits	Mean *TimeInImage*
		C	1845	49.7%	1.6
	B		684	18.4%	2.7
A			626	16.9%	2.0
A		C	327	8.8%	2.4
A	B		131	3.5%	3.2
	B	C	95	2.6%	4.0
A	B	C	5	0.1%	10.8
Total			3713	100.0%	2.1

Table S5: Image visits in right image by subphase(s). n=3713 Comparison-phase right image visits.

TimeInImage is calculated on each image visit, and its value is associated with each fixation within that image visit. Visits to the right image tend to be much longer in subphase B than in subphase C (Fig. S36).

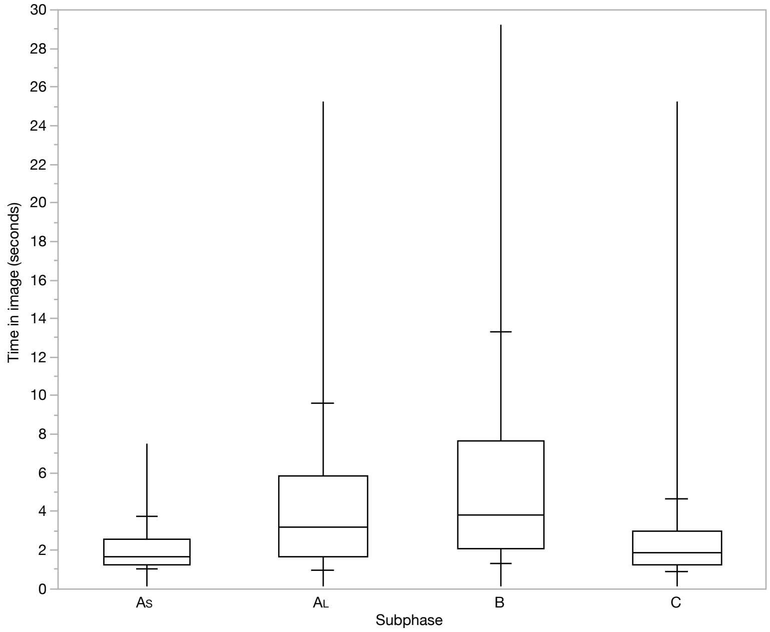

Fig. S36: Distribution of the *TimeInImage* metric by subphase. Medians: A_S 1.7 seconds; A_L 3.2; B 3.8; C 1.9. n=22747 right-image fixations. Crossbars indicate deciles. (Although the threshold for A_S is a period of less than 1 second prior to the first fixation, TimeInImage is usually greater than that, indicating that there was a period of less than 1 second outside the target prior to the examiner looking in the target.)

Appendix SI-13.4 DetailedBackAndForth metric

We defined the *DetailedBackAndForth* metric to measure multiple returns to approximately the same small location in an image after a left-right (or right-left) transition. *DetailedBackAndForth* is a count of image visits (not individual fixations): for example, if the examiner looks at a relatively small area in the right image, looks at the left, and looks back at the same area in the right image, *DetailedBackAndForth* is set to 1, and is incremented after every consecutive left-right transition to the same location; it is reset to 1 when the examiner looks in a different location. An examiner is said to have returned to the "same small" location if the centroids of the fixations within each sequence are within 88 pixels and no two fixations within either sequence are further than 176 (i.e. 2*88) pixels apart.

88 pixels was used because a circle with a 88 pixel radius is about the same area as the 150x150 target. We also used 44 pixels instead of 88, with similar results but fewer nonzero values and smaller values.

DetailedBackAndForth is calculated over a series of image visits, and its value is associated with each fixation within those image visits. Fig. S37 and Fig. S38 show the association of *DetailedBackAndForth* and subphase: although a zero value does not tell us anything about subphase, a non-zero value (especially three or more) is strongly associated with subphase B or C.

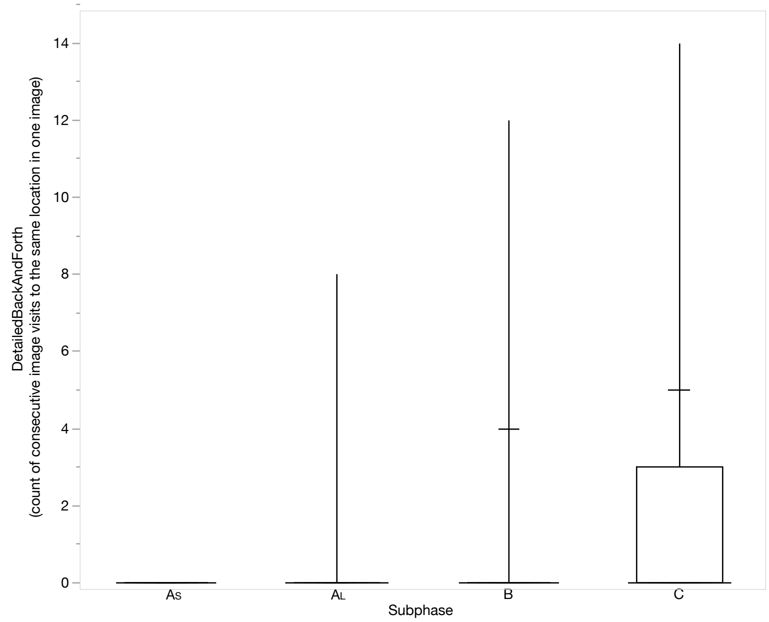

Fig. S37: Distribution of the *DetailedBackAndForth* metric by subphase. All medians are 0. n=22747 right-image fixations. Crossbars indicate deciles.

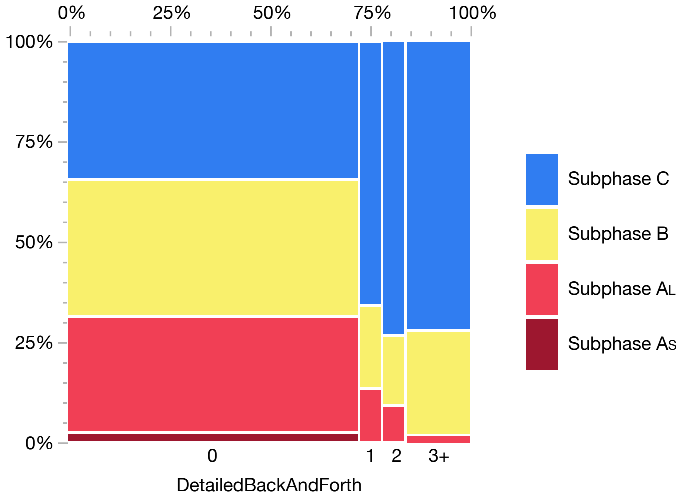

Fig. S38: *DetailedBackAndForth* by subphase. n=22747 right-image fixations.
